# pH Regulates Ion Dynamics in Carboxylated Mixed Conductors

**DOI:** 10.1021/acs.chemmater.5c03288

**Published:** 2026-02-09

**Authors:** Zeyuan Sun, Mengting Sun, Rajiv Giridharagopal, Robert C. Hamburger, Siyu Qin, Haoxuan Li, Mitchell Hausback, Yulong Zheng, Bohyeon Kim, Heng Tan, Thomas E. Gartner, Elizabeth R. Young, Christopher J. Takacs, David S. Ginger, Elsa Reichmanis

**Affiliations:** † Department of Chemical and Biomolecular Engineering, 1687 Lehigh University, Bethlehem, Pennsylvania 18015, United States; ‡ Department of Chemistry, 7284 University of Washington, Seattle, Washington 98195, United States; § Department of Chemistry, Lehigh University, Bethlehem, Pennsylvania 18015, United States; ∥ School of Chemistry and Biochemistry, 1372 Georgia Institute of Technology, Atlanta, Georgia 30332, United States; ⊥ Department of Computer Science and Engineering, 1687 Lehigh University, Bethlehem, Pennsylvania 18015, United States; # Stanford Synchrotron Radiation Lightsource SLAC National Accelerator Laboratory, Menlo Park, California 94025, United States

## Abstract

Coupled ionic and electronic transport underpins processes as diverse as electrochemical energy conversion, biological signaling, and soft adaptive electronics. Yet, how chemical environments such as pH modulate this coupling at the molecular scale remains poorly understood. Here, we show that the protonation state of carboxylated polythiophenes provides precise chemical control over ion dynamics, doping efficiency, solvent uptake, and mechanical response. Using a suite of multimodal *operando* techniques, supported by simulations, we reveal that pH dictates the balance of cation/anion uptake during electrochemical doping. Mapping across pH uncovers a quasi-nonswelling regime (≈pH 3–3.5) where charge compensation proceeds with minimal volumetric change yet pronounced stiffening. These findings establish molecular acidity as a general strategy to program ionic preference and mechanical stability, offering design principles for pH-responsive mixed conductors and soft electronic materials that couple ionic, electronic, and mechanical functionality.

## Graphical abstract

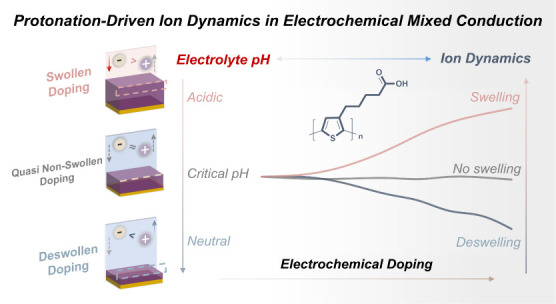

## 1 Introduction

Electronic functionality has expanded far beyond computation and information storage to encompass intelligent, adaptive systems that engage directly with their environments, including living organisms.^1^ Bioelectronics, which bridges electronic and biological domains through devices capable of transducing electrical and biochemical signals bidirectionally, has emerged as a cornerstone of this evolution.^2,3^ Such systems hold promise across diverse application spaces—from energy storage^4,5^ and neuromorphic computing^6,7^ to neurotransmitter regulation^8^ and tissue-integrated sensing and actuation,^9,10^ where ionic species serve as key mediators of signal and charge transport.

Among these ionic species, protons (H^+^) occupy a uniquely central role. Local proton concentration (pH) governs charge transport in biological media and modulates fundamental processes, including neuronal signaling,^11,12^ enzymatic catalysis,^13^ and cellular metabolism.^14^ Thus, pH is not a passive descriptor but an active regulatory parameter, dynamically shaping protein conformation,^15^ enzyme activity,^16^ and ion channel behavior.^17^ Local gradients, whether at inflamed tissues, tumor microenvironments^18^ or neural synapses,^19^ are tightly correlated with physiological and pathological states. Consequently, pH responsiveness is a fundamental building block for materials and devices to seamlessly interface with biological systems. Integrating such responsiveness into synthetic systems remains challenging. Unlike metal ions, protons often migrate through dynamic hydrogen-bonded networks, complicating transport modeling and experimental quantification.^20^ Furthermore, proton-coupled electron transfer (PCET) processes are highly sensitive to local pH and hydration,^21^ often leading to nonlinear, poorly understood redox behavior.^22^ Disentangling the contributions of protons from coexisting cations remains a persistent hurdle. These challenges are compounded by the limited availability of suitable materials and/or *operando* techniques capable of directly resolving pH-induced activity at buried interfaces. Thus, designing materials that enable selective, stable, reversible pH responsiveness in complex media requires a deeper mechanistic understanding of coupled ionic/protonic-electronic dynamics across multiple length and time scales.

Organic mixed ionic–electronic conductors (OMIECs) provide a versatile platform for investigating coupled ionic–electronic transport across both biological and energy interfaces.^23^ OMIECs can accommodate a broad range of ionic species—from physiological ions such as Na^+^, K^+^, and Cl^–^ to energy-relevant species including Li^+^, TFSI**^–^**, and PF_6_^–^. Given the centrality of pH in regulating functionality, materials designed for bioelectronic or iontronic interfaces must tolerate and respond predictably to local pH, requiring molecular architectures capable of translating subtle acid–base equilibria into controlled ionic/electronic behavior. Such responsiveness has been reported in certain OMIECs; however, most studies interpret device-level performance shifts as indirect indicators rather than direct probes of underlying molecular mechanisms.^24–27^ Elucidating how pH-induced structural and electrochemical changes evolve within complex ionic environments is essential to inform rational materials design and unlock new opportunities for engineering of OMIECs for emerging applications.

Here, we demonstrate that pH fundamentally reshapes the electrochemical doping mechanism in p-channel carboxylated OMIECs, governing their doping efficiency, ion/water dynamics, and structural evolution. The protonation–deprotonation equilibrium of the carboxylic acid side chains imparts finely tunable fixed charges with pH changes, while hole conduction along the conjugated backbone is balanced primarily by anion compensation. This configuration allows pH to act as a control that modulates ionic–electronic coupling without direct proton involvement, enabling pH-related process studies. Utilizing multimodal *operando* characterization techniques and coarse-grained (CG) molecular dynamics (MD) simulations, we reveal how pH regulates ion dynamics, leading to a cascade of structural, spectroscopic, and electrochemical transformations. Collectively, these findings establish carboxyl moieties as powerful design motifs, advancing the development of robust, pH-responsive materials for next-generation bioelectronic and multistimuli interfaces and beyond.

## 2 Results and Discussion

### 2.1 pH-Dependent and pH-Independent Mixed Conduction

To probe the pH-responsive electrochemical behaviors, poly­[3-(4-carboxylbutyl)­thiophene-2,5-diyl] (P3CBT-P) served as a model carboxylated conjugated polyelectrolyte.^28,29^ Electrochemical quartz crystal microbalance with dissipation monitoring (EQCM-D) measurements were conducted in both neutral and acidic aqueous electrolytes. As shown in Figure S1–S2, neither the specific identity of the noncompensating ion (cations for p-channel doping) nor moderate variations in compensating ion concentration (anions in p-channel doping) alone account for the distinct electrochemical response, ruling out extrinsic factors and underscoring the intrinsic pH sensitivity of COOH functionality. Under neutral pH, EQCM-D reveals a net mass *loss* during electrochemical doping (Figure 1a), consistent with prior observations of cation-carboxylate binding that drives deswelling as cation release outweighs anion uptake.^29^ In contrast, acidic conditions elicit a dramatically different response, with pronounced mass *gain* during doping, reflecting a shift in the ion exchange dynamics. We hypothesize that suppressed carboxyl dissociation at low pH weakens cation binding affinity, enabling anion uptake to dominate the swelling/gravimetric process.

**Figure 1:**
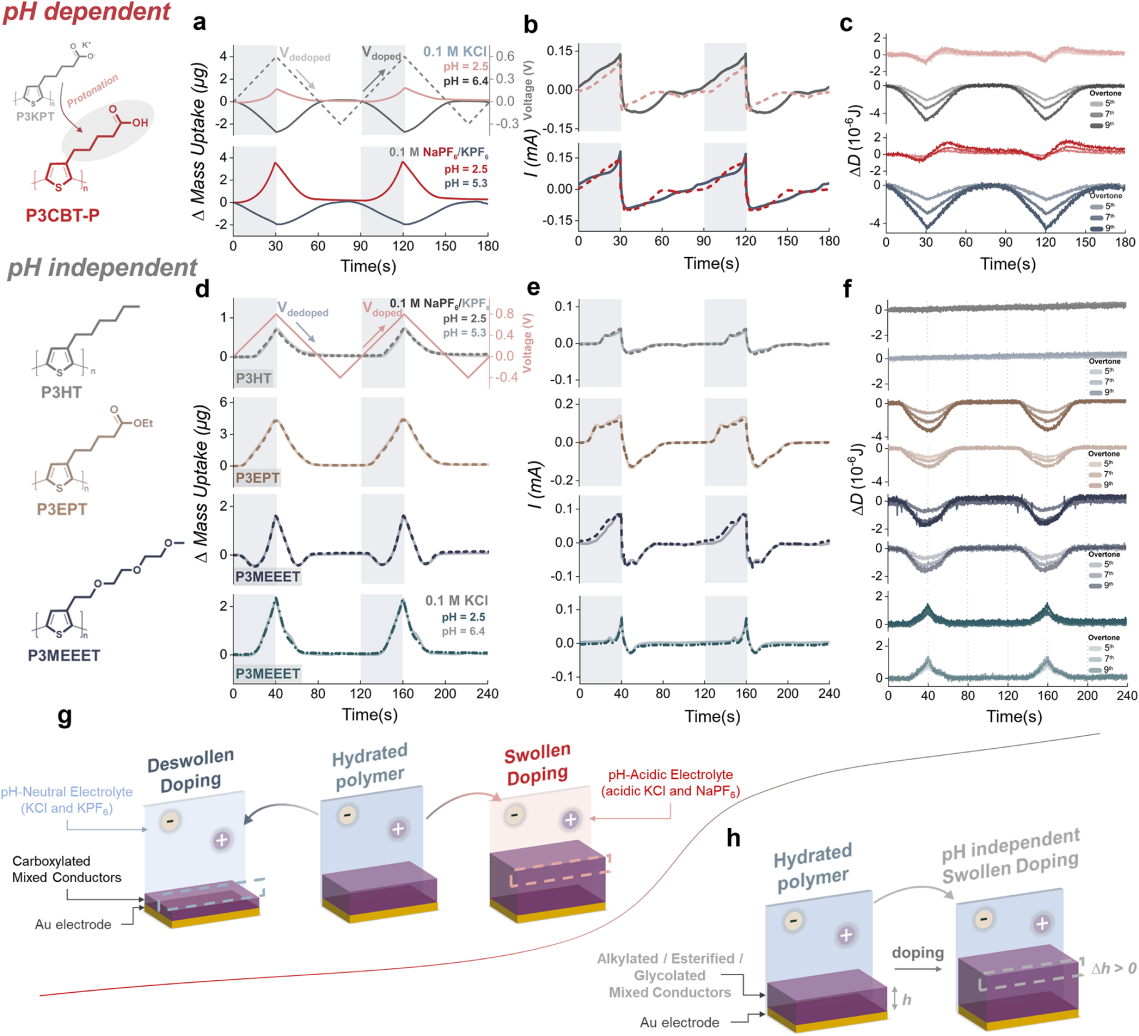
pH-dependent and pH-independent mixed conduction in polythiophene-based OMIECs. Chemical structures and pH-modulated electrochemical quartz crystal microbalance with dissipation (EQCM-D) measurements of the studied polymers. Measurements were conducted in both neutral and acidic aqueous electrolytes, including 0.1 M KCl (pH 6.4), 0.1 M KPF_6_ (pH 5.3), 0.1 M KCl acidified to the designated pH with HCl, and 0.1 M NaPF_6_ (pH 2.5). The **top panel** presents the **pH-dependent** group, featuring protonated carboxylic acid-functionalized polythiophene (P3CBT-P) derived from as-cast P3KPT. Corresponding electrochemical measurements include (a) mass change/voltage profiles (9th overtone), (b) cyclic voltammetry-derived current response, and (c) dissipation energy variation in neutral and acidic electrolytes. The **bottom panel** illustrates the **pH-independent** functional group, consisting of P3HT, P3EPT, and P3MEEET, with their corresponding (d) mass change/voltage profiles, (e) cyclic voltammetry-derived current response, and (f) dissipation energy variation in the same electrolytes, as the carboxylated mixed conductors. (g) Schematic representation of the tuning principle for **pH-dependent mixed conduction**, where swelling behavior is modulated by pH—neutral pH favors deswollen doping, while acidic pH promotes swollen doping. (h) Schematic depiction of **pH-independent mixed conduction**, where swollen doping occurs, irrespective of pH variations.

Corresponding EQCM-derived voltammograms (Figure 1b) reveal a pronounced shift in behavior, where acidic conditions delay the onset of oxidation relative to neutral pH by ∼0.2 V. Supporting evidence from passive swelling (Figure S3) and contact angle measurements (Figure S4) confirms pH-switchable wettability, where increased protonation reduces hydrophilicity, thus affecting ion–polymer interactions in an ion-dependent manner. Notably, a higher degree of protonation leads to less swelling (∼8% vs ∼16%, respectively) under unbiased conditions.^30,31^ Voltage-dependent dissipation profiles (Figure 1c) provide further evidence of altered ion–polymer interactions, where acidic conditions produce a biphasic mechanical response—an initial stiffening in the doping range followed by softening during dedoping—distinguished from the reversible rigidity observed at neutral conditions, indicating dynamic reorganization of the polymer network during doping. These results collectively point to pH-regulated modulation of polymer hydrophilicity, governed by the COOH protonation state and associated transient H-bonding and viscoelasticity.

To establish that pH-responsive behavior is intrinsically linked to the presence of COOH moieties, we performed pH-modulated EQCM-D measurements on a series of polythiophene-based OMIECs, namely, poly­(3-hexylthiophene-2,5-diyl) (P3HT), poly­[3-(ethyl-5-pentanoate)­thiophene-2,5-diyl] (P3EPT), and poly­(3-[2-[2-(2-methoxyethoxy)­ethoxy]­ethyl]­thiophene-2,5-diyl) (P3MEEET) under Cl^–^ and PF_6_^–^ electrolyte environments at varied pH (Figure 1d,e). These *non*-ionizable polymers exhibited *no* appreciable variation in mass change, electrochemical response, or viscoelastic dissipation across pH environments, confirming that *ionizable,* COOH-bearing side chains are essential for enabling proton-mediated doping dynamics. The striking transition observed in P3CBT-P, from the deswollen doping state at neutral pH to the highly swollen state under acidic conditions, highlights the criticality of pH in reconfiguring ion–polymer and polymer–polymer interactions, fundamentally altering the electrochemical doping mechanism (Figure 1g). In contrast, nonionizable analogues retain anion-dominated, pH-invariant swollen doping behavior (Figure 1h).

CG-MD simulations of single P3CBT chains in aqueous KCl directly probed ion–polymer interactions as a function of the side chain protonation state (Figure 2a). Simulations were performed in fully protonated (low pH) and fully deprotonated (high pH) states (see Methods and SI-Discussion 1 and Table S1–S3). In direct correspondence with the experimental hydrophilicity results above, deprotonated P3CBT showed extended configurations, while those of the protonated analog were collapsed, reflecting increased hydrophilicity in the deprotonated system. Radial distribution functions, *g*(*r*), also support the above hypotheses around pH effects on polymer–ion and polymer–water interactions (Figure 2b,c). Namely, deprotonated COO^–^ groups promote side chain-cation correlations and suppress backbone-anion correlations relative to COOH. The polymer–water *g*(*r*) also allows us to analyze the solvation behavior of the chains. The polar side chain–water *g*(*r*) exhibits a pronounced first peak (Figure 2b), indicating strong solvation of the polar side chain bead (COOH/COO^−^). In contrast, the thiophene–water *g*(*r*) shows a weaker first peak (Figure 2c), indicating that the conjugated backbone remains poorly solvated and that hydration is dominated by side chain–water interactions. Upon increasing pH (deprotonation), the polar side chain–water correlations are enhanced even further, consistent with more favorable water–polymer interactions.

**Figure 2:**
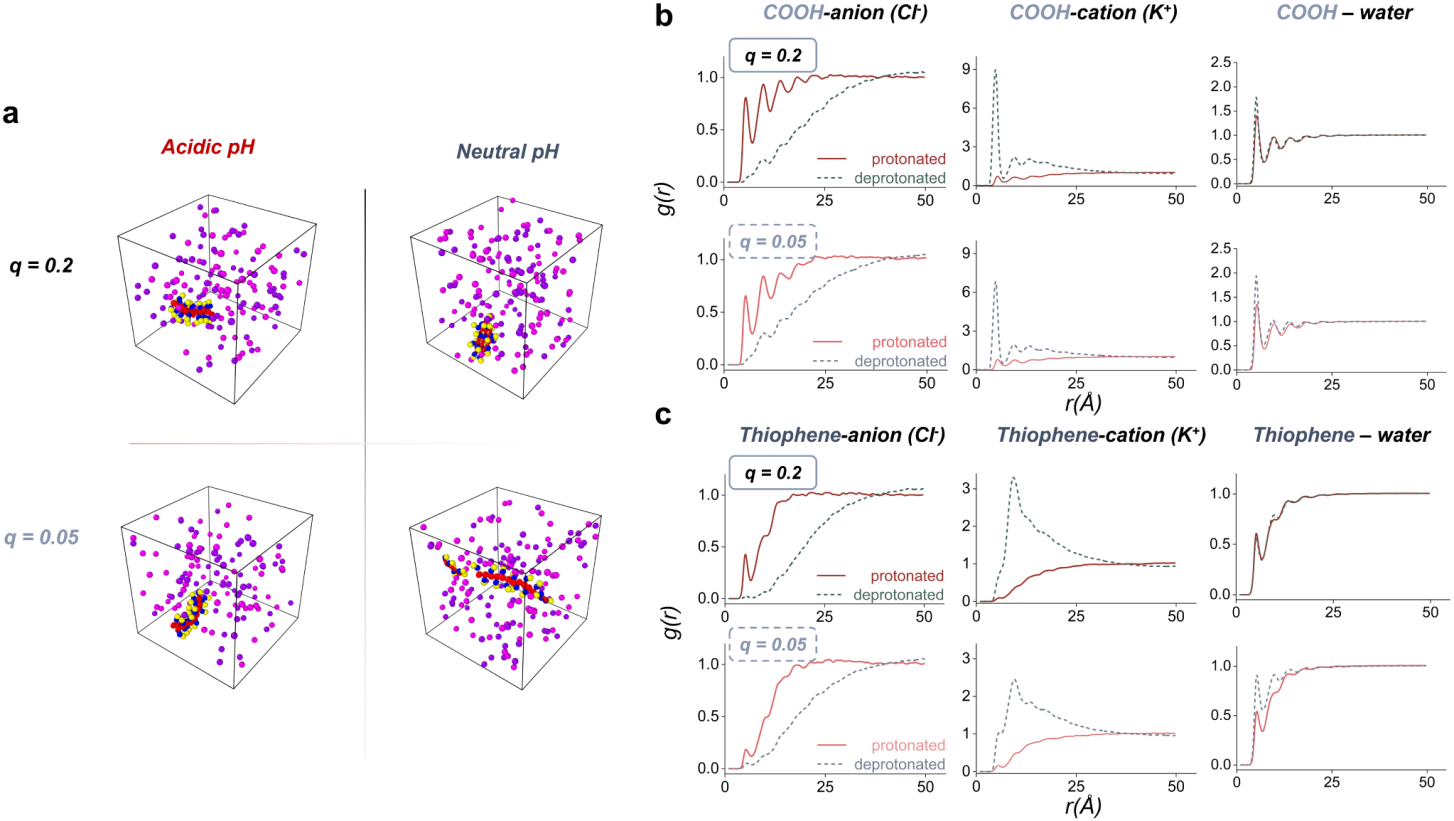
**a)** Coarse-grained molecular dynamics (MD) snapshots of a single P3CBT chain with different backbone charge densities (*q* = 0.2 and 0.05 per monomer) simulated in aqueous KCl under acidic (protonated) and neutral (deprotonated) pH conditions. Water molecules are hidden for clarity. **b)** Radial distribution functions (g­(r)) between carboxylic acid groups and surrounding species (Cl^–^, K^+^, and water), and **c)** g­(r) between thiophene backbones and the same species.

This pH responsive behavior appears robust across processing conditions: P3CBT-P and P3CBT (obtained from DMSO solutions of P3CBT) both exhibit pH-dependent mass uptake, electrochemical response, and OECT performance (Figure 3a–c). Notably, the degree of COOH protonation strongly influences electrochemical behavior, with earlier onset potentials observed under a highly deprotonated (pH 6–9) vs highly protonated (pH 2.5) state. Mass transport kinetics (Figure 3d,e) reveal a consistent trend across both polymers with gravimetric response that is slower under pH acidic conditions. Despite the reduced swelling associated with DMSO processing, the results align with increased hydrophobicity and altered doping dynamics in the protonated state. Together, these comparisons highlight the unique pH sensitivity imparted by COOH and establish a design strategy for modulating mixed conduction via control of local proton concentration.

**Figure 3:**
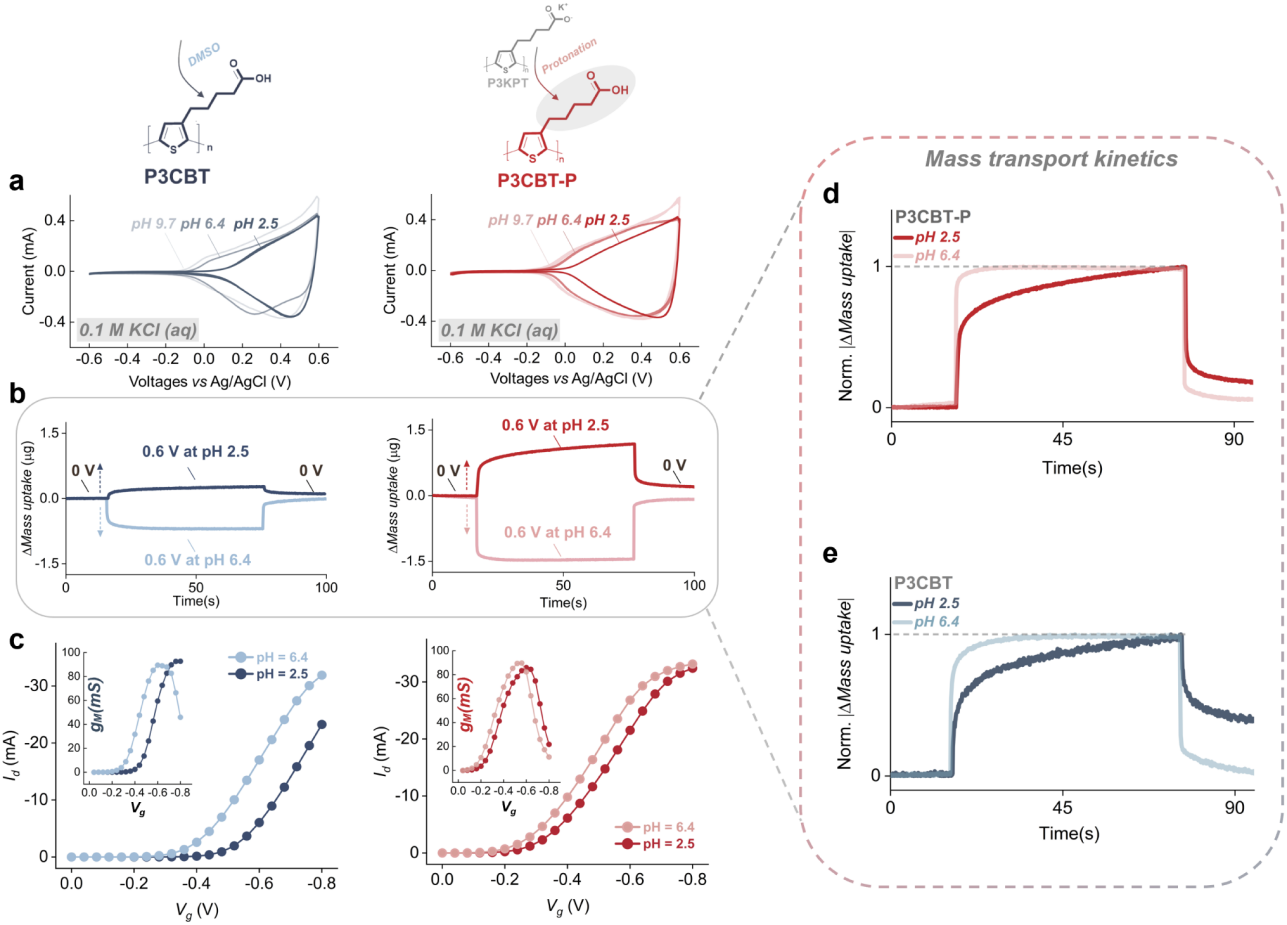
Side-by-side comparison of P3CBT (DMSO) and P3CBT-P (derived by protonation method) under varying pH conditions. **a)** cyclic voltammetry, **b)** real-time gravimetric behavior by EQCM-D, and **c)** interdigitated OECT (iOECT) transfer curves in 0.1 M KCl at neutral and acidic pH. Alkaline conditions in **a)** were not further explored due to complications arising from acid–base neutralization and instability issues that potentially dissolve the polymers. Normalized mass transport kinetics (from **b**) during electrochemical equilibrium highlight differences in mass transport kinetics at different pH conditions for **d)** P3CBT-P and **e)** P3CBT, respectively.

### 2.2 Real-Time Probing of Ionic and Structural Reorganization with *Operando* X-ray Scattering and Scanning Probe Techniques

To further explore the impact of pH on ion dynamics, *operando* grazing incidence X-ray fluorescence (GIXRF) enabled tracking of ion movement in and out of the bulk thin film during electrochemical cycling. Analysis focused on the potassium K-edge emission (∼3.4 keV), a direct probe of cation concentration in the carboxylated polythiophenes.^29^ All K^+^ signals were normalized to the elastic scattering peak, enabling direct qualitative comparison between environments (Figure S5–S6). GIXRF spectra (Figure 4a) show pH-dependent potassium intensity modulation. At pH 6.4, the signal exhibits pronounced cyclic oscillations, indicating reversible expulsion/reinsertion of K^+^ during (de)­doping—consistent with a cation-compensated mechanism facilitated by deprotonated COOH groups. In contrast, at pH 2.5, the K^+^ signal is weaker with a reduced magnitude, suggesting diminished cation involvement and a shift toward anion-dominated doping. Notably, after dedoping at pH 6.4, the K^+^ intensity increases, indicating net cation accumulation within the polymer, a trend not observed under acidic conditions. These observations demonstrate that the protonation state of the COOH governs cation transport, even within the highly swollen, acidic regime. Complementary liquid-phase atomic force microscopy (AFM) with bias applied *in situ* under identical pH conditions (Figure 4b) corroborates coupling between mass and dimensional change: doping at neutral pH yields a gradual thickness *decrease* up to ∼28 nm (**−8%**), whereas acidic conditions produce a gradual *increase* (∼37 nm, **+15%**) (Figure S7). These results confirm the hypothesis that reduced cation-polymer binding at low pH drives a transition toward anion-dominated, swollen states.

**Figure 4:**
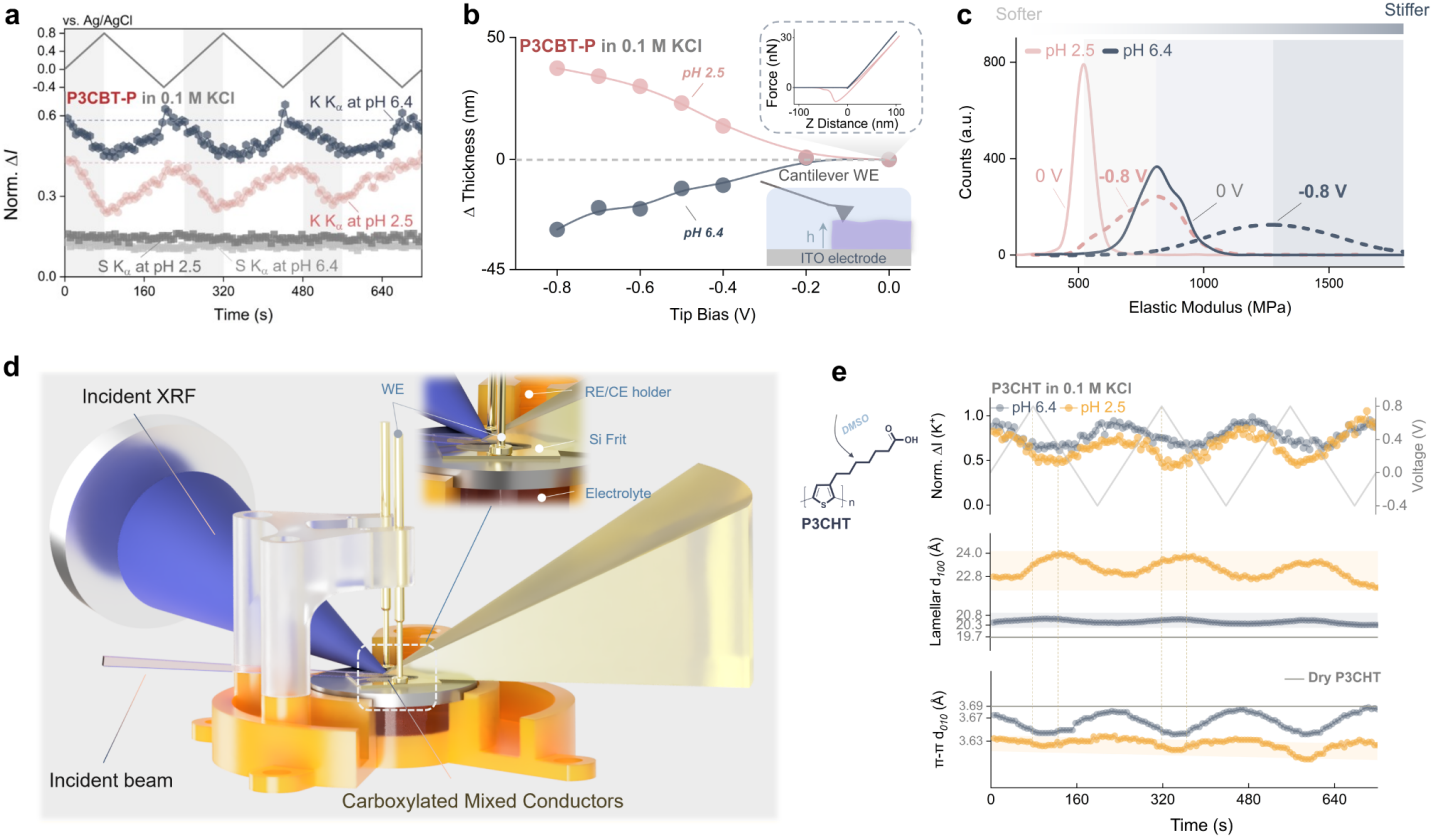
Multimodal *operando* characterization of carboxylated polythiophenes. a) *Operando* grazing incidence X-ray fluorescence (GIXRF) of P3CBT-P in 0.1 M KCl at pH 6.4 and pH 2.5. b) *In situ* AFM thickness tracking and AFM nanoindentation during 0 V potential holds at pH 6.4 and pH 2.5. c) *In situ* force-mapping under the same electrolyte and pH conditions. d, e) Combined *operando* apparatus and results for grazing incidence wide-angle X-ray scattering and X-ray fluorescence (GIWAXS + GIXRF) of P3CHT in 0.1 M KCl at pH 6.4 and pH 2.5.

Further evidence of stiffness correlated with electrochemical strain was demonstrated *in situ* (Figure 4c), where COOH functionalization shows a lower elastic modulus (∼520 MPa) in acidic vs neutral (∼800 MPa) pH at 0 V, likely owing to complex protonation–hydration interactions.^32,33^ Upon doping (−0.8 V), both pH conditions lead to pronounced stiffening, with the elastic moduli increasing to 800 MPa at pH 2.5 and ∼1250 MPa at pH 6.4. This trend is consistent with the reduced dissipation observed in Figure 1c and is reflective of doping-induced structural and electrostatic effects. In both cases, doping injects anions while expelling cations previously associated with fixed COO^–^ groups. Ion exchange reduces internal hydration, collapses free volume, and eliminates the plasticizing effect.^34^ Plausibly, pH-modulated ionic cross-linking during doping outweighs plasticization,^35^ and additional anion-COO^–^ repulsion may further restrict chain mobility, enhancing stiffness. These effects transform doping from a softening mechanism^36^ (common in glycolated polymers) into a stiffening one, highlighting a distinct structure-mechanics coupling in carboxylated OMIECs.

To further study the bulk thin film ion content structural dynamics in the pH-induced changes, we used *operando* grazing incidence wide-angle X-ray scattering (GIWAXS) with simultaneous GIXRF collection (Figure 4d). Although P3CBT-P remains the benchmark carboxylated mixed conductor across the series, the longest side chain carboxylhexyl variant, P3CHT, exhibits well-defined *ex-situ* GIWAXS features (Figure S8–S9). This material retains a pH-dependent swelling response (Figure S10), making P3CHT ideal for *operando* GIWAXS–GIXRF studies. Figure 4e summarizes the evolution of the P3CHT elemental and structural response during CV at pH 6.4 and 2.5 in 0.1 M KCl. GIXRF reveals reversible K^+^ insertion/expulsion. The GIWAXS lamellar (100) reflection remains nearly unchanged at pH 6.4 (Δ*d* ≈ 0.6 Å, 3%) but is pre-expanded by ∼3.1 Å (15%) at pH 2.5, indicating substantial ionic intercalation prior to cycling. Subsequent doping at pH 6.4 induces minimal expansion (∼0.5 Å), consistent with K^+^ expulsion (deswollen doping), whereas pH 2.5 exhibits an ∼1.2 Å expansion and pronounced structural hysteresis aligned with the electrochemical delay. The π–π (010) peak contracts slightly under both conditions (<0.1 Å), suggesting tighter yet intact backbone packing. These results indicate highly anisotropic electrochemical swelling, confined primarily to side-chain lamellae, while the conjugated cores remain structurally and electronically coupled throughout.

Together, *operando* GIWAXS–GIXRF results reveal a coherent picture of the pH-dependent ion distribution in carboxylated OMIECs. In crystalline domains, the behaviors of cation/anion pairs exhibit pronounced pH dependence, with anions playing the dominant structural role. While doping induces lamellar expansion and π–π contraction, the extent of expansion is minimal at neutral pH but pronounced under acidic conditions. Protons are likely synchronized with cation motion under acidic conditions.^37^ Correlating this trend with macroscopic swelling reveals that ionic species primarily occupy amorphous domains, where pH-dependent, spatially heterogeneous volumetric change occurs.^38^ This localization rationalizes the coupled structural and mechanical responses, indicating that the amorphous matrix governs volumetric dynamics, while crystalline order sustains electronic transport.

### 2.3 Protonation-Mediated Modulation of Charge Delocalization and Doping

To elucidate the pH-dependent modulation of charge carrier dynamics further, *operando* ultraviolet–visible–near-infrared (UV–vis–NIR) spectroelectrochemistry was performed on P3CBT-P (Figure 5a,b). Across all electrolyte conditions, discrete electronic states are revealed: a neutral-like configuration under reducing bias (−0.6 V), a doped state under oxidative bias (+0.8 V), and an intermediate “self-doped” state at open circuit potential (OCP).^39,40^ This OCP feature reflects a preexisting population of charge carriers and/or ionic species within the polymer, suggesting intrinsic predoping behavior in the absence of applied bias (Figure S11).^41^ Upon anodic polarization, systematic attenuation of the ∼530 nm absorption is accompanied by the emergence of polaron (∼880 nm) and bipolaron (∼1350 nm) bands, consistent with oxidative doping and the progressive delocalized charge carrier formation. Voltage-dependent spectral evolution (Figure 5c) exhibits a clean, monotonic trend, underscoring electrochemical control over charge carrier formation. Acidic electrolytes induce a noticeable delay in polaron and bipolaron growth onset vs neutral conditions: Cl^–^-based systems show a more pronounced lag than PF_6_^–^. Nevertheless, at higher potentials (≥0.7 V), films doped under acidic pH exhibit elevated absorption intensities across all doping bands, indicating a higher final doping level. These results suggest that pH governs ion exchange kinetics and carrier formation, enabling access to deeper doping regimes in carboxylated polythiophenes—potentially via enhanced anion uptake with reduced electrostatic repulsion.

**Figure 5:**
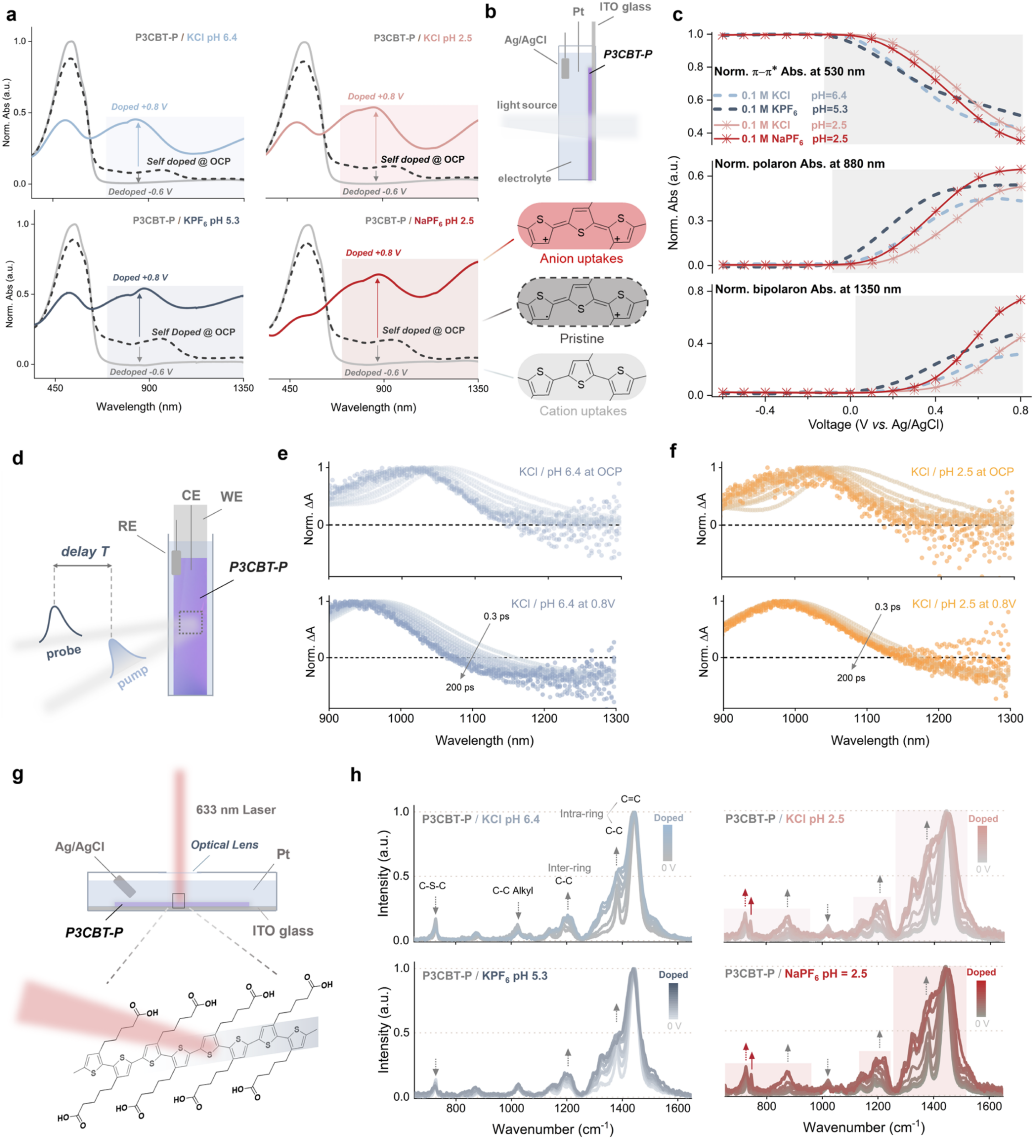
pH-dependent photophysical characterization of P3CBT-P. a) Spectroelectrochemistry of P3CBT-P in pH neutral (0.1 M KCl, pH 6.4; 0.1 M KPF_6_, pH 5.3) and pH acidic (0.1 M KCl, pH 2.5; 0.1 M NaPF_6_, pH 2.5) electrolytes. b) Schematic of the *in situ* spectroelectrochemical cell. c) The evolution of steady-state absorbance during electrochemical doping from −0.6 to 0.8 V (vs Ag/AgCl), showing changes in the 0–1 neutral (530 nm), polaron (880 nm), and bipolaron (1350 nm) peaks. d) Schematic of the *operando* transient absorption spectroscopy (TAS) setup and the doped-state (0.8 V). TAS spectra at e) pH neutral and f) pH acidic KCl conditions at open circuit potential and doping potential 0.8 V vs Ag/AgCl. g) *Operando* Raman experiment setup and h) evolved spectrum for P3CBT-P in neutral and acidic electrolytes from 0 V to 0.8 V doped condition.

To further probe the nature of photoexcited charge carriers under varying pH conditions, *operando* femtosecond transient absorption spectroscopy (TAS) was performed. In neutral KCl, the spectra exhibit a well-defined photoinduced absorption (∼1000 nm), with an onset near 940 nm (Figure S12–S13). The dynamics show rapid decay components on the subnanosecond time scale (Figure S14, ∼0.1–0.2 ns), consistent with polaronic relaxation processes in moderately doped conjugated systems (Figure 5e).^42^ At acidic pH, TAS reveals a broader red-shifted band, accompanied by similarly fast decay. Generally, the red-shifted and faster-decaying polaron spectra in doped systems indicate more delocalized charge carriers, in agreement with GIWAXS evidence of reduced π–π stacking distance at lower pH (Figure 5f).

*Operando* Raman spectroscopy explored the molecular-level structural changes underlying pH-modulated doping (Figure 5g). Across all conditions, a progressive evolution of vibrational features is observed upon doping (Figure 5h), notably in the C=C stretching regions, reflecting changes in backbone conjugation and planarity.^43^ As the applied potential increases, the quinoid-like C=C symmetric stretch intensity grows significantly, accompanied by a redshift with significantly higher *I*_c–c_/*I*_c=c_ under lower pH indicating increased planarization and electronic delocalization.^28,44^ At lower wavenumbers (700–1000 cm^–1^) associated with ring deformation and side chain interactions, pH-dependent differences emerge. Under acidic conditions, new bands appear, suggesting the formation of distinct structural motifs or ion–polymer interactions—conceivably arising from enhanced anion uptake or stronger electrostatic coupling between protons and carboxylate groups. Collectively, the UV–vis–NIR, TAS, and Raman results reveal that pH modulation both tunes the doping level and reshapes the charge carrier electronic configuration and relaxation dynamics. These *operando* spectroelectrochemical insights establish that pH-regulated ion exchange governs carrier delocalization and the overall optoelectronic character of carboxylated polythiophenes. Taken together, a complete mechanistic picture emerges, revealing pH-dependent ion preference and dynamics in carboxylated OMIECs, as shown in Figure 6. Carboxylic acid functionalization introduces a small density of intrinsic charge carriers, even without applied bias. At neutral pH, deprotonation of carboxylic acid groups generates negatively charged −COO^–^ sites that preferentially attract cations from the electrolyte while partially excluding anions. Under acidic conditions, protonation reduces the population of −COO^–^ groups, leading to weaker cation association but increased anion uptake (see SI-Discussion 2, Table S4, Figure S15) During electrochemical doping, cation expulsion outweighs anion uptake, yielding a deswollen doping mode with a limited doping level. Under acidic conditions, protonation reduces the fixed charge density, weakening the cation affinity while allowing greater anion ingress. The diminished Donnan exclusion at low pH leads to a swollen doping mode, reaching higher oxidation states at elevated bias.

**Figure 6:**
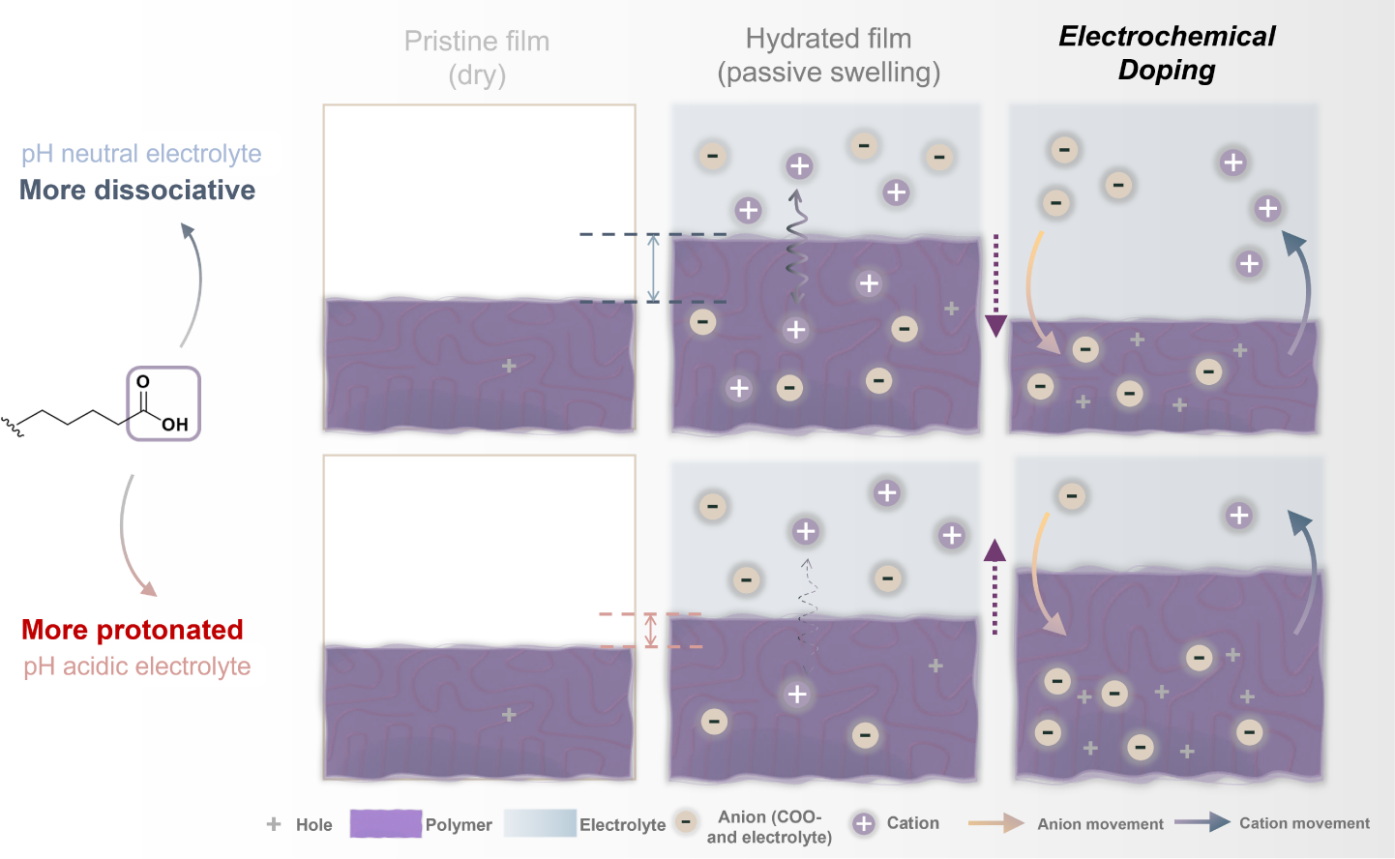
Schematic of pH-dependent ion dynamics in carboxylated mixed conductors.

### 2.4 Macroscale and Microscale Insights into Quasi-Non-Swelling Behavior at the Critical pH in Carboxylated Mixed Conductors

As the pH-dependent behavior was consistently confirmed across *operando* measurements, we extended the investigation to finer pH resolution. P3CBT was characterized in 0.1 M KCl across a range of conditions from neutral to increasingly acidic (Figure 7a,b). Pronounced deswelling occurs upon doping at pH 5.5, while decreased pH leads to progressive reduction in mass change, indicating that increased protonation suppresses deswelling. A critical point emerges near pH 3.0–3.5 where the gravimetric response becomes minimal: mass uptake decreases by approximately 85% and 95%, respectively, for thin and thick films. To verify this quasi-nonswelling state, we performed *in situ* AFM with an applied bias under the same conditions, revealing only ∼2.5 nm thickness change during doping—corresponding to less than 0.7% of the initial film thickness (Figure 7c). A similar effect was observed in P3CBT-P at pH 3.5, where the thickness change was limited to <0.3%, further supporting the presence of a volumetrically stable, nanoscopic doping mode at the critical pH.

**Figure 7:**
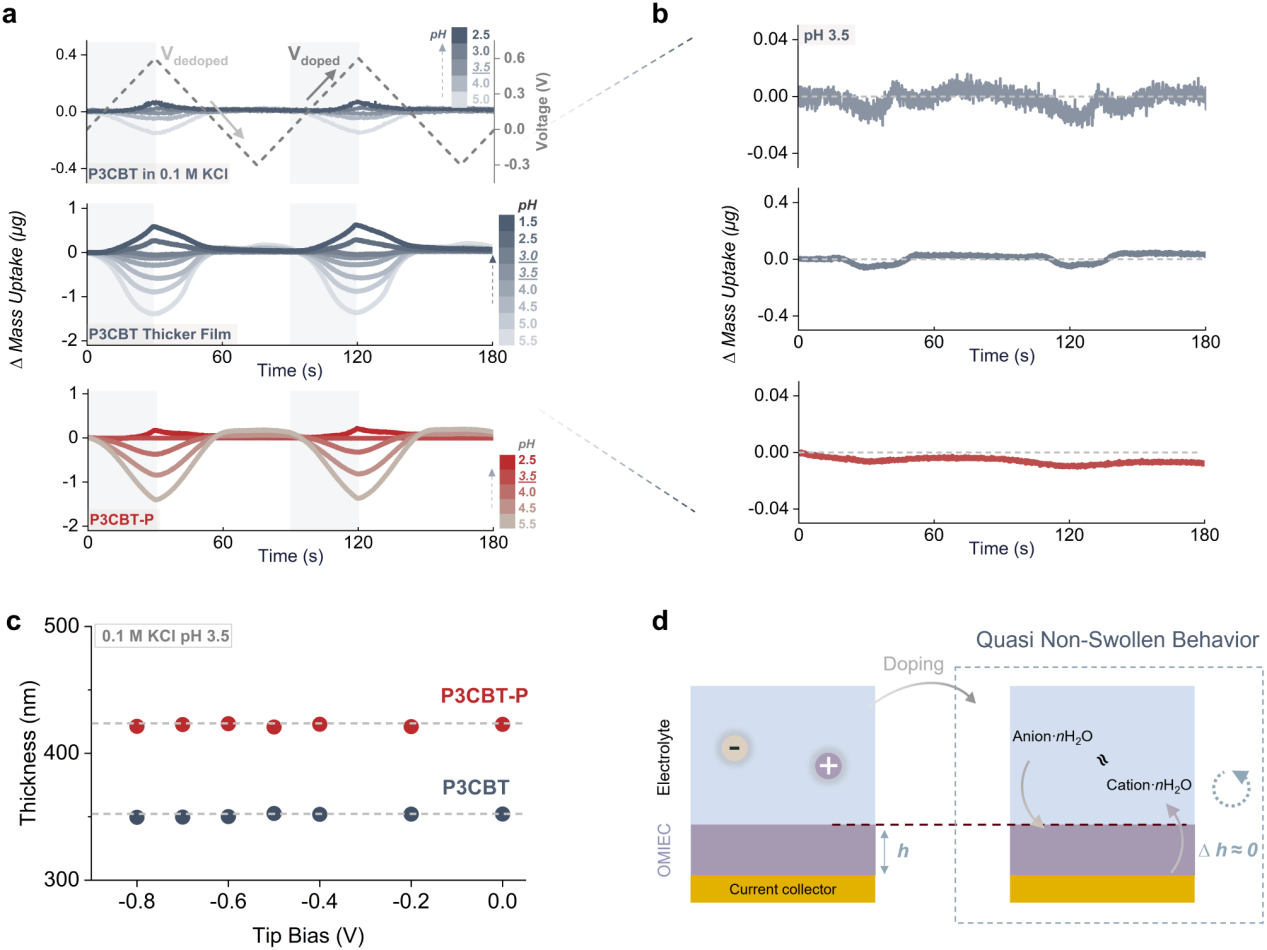
Critical pH enables quasi-nonswelling behavior for carboxylated mixed conductors. a) pH-dependent gravimetric response of P3CBT and P3CBT-P in 0.1 M KCl, spanning pH neutral to acidic conditions, confirming that the quasi-nonswelling regime is process-independent across solvents and governed by the carboxylic acid functionalization. b) Magnified view near the critical pH of 3.5, revealing a nonmonotonic swelling trend; c) *in situ* AFM confirming volumetrically stable film thickness under electrochemical doping at the critical pH. d) Schematic illustration of the quasi-nonswelling mechanism.

These findings establish that a quasi-nonswelling doping mode can be achieved at a critical pH for carboxylated OMIECs. This volumetrically stable condition likely reflects a charge-compensation regime where the number of hydrated anions entering the film is balanced by hydrated cations and protons exiting, minimizing macroscale structural expansion despite ongoing redox activity (Figure 7d). We propose that heterogeneous, bidirectional cation–anion transport during electrochemical doping drives compensating responses between crystalline and amorphous domains at the critical pH, resulting in minimal macroscopic volume and mass change. This state is linked to the protonation equilibrium of the COOH groups, suggesting that it arises from an electrochemically balanced dissociative regime unique to COOH-functionalized polymers. The precise pH at which this balance occurs, however, is influenced by environmental factors, including ion identities, electrochemical window, and polymer effective p*K*_a_, which modulate the onset of volumetric stability. The pH-responsive behavior driven by protonation originates from the presence of ionic pendant groups. Consequently, in principle, other materials functionalized with ionic moieties—whether acidic, zwitterionic, or basic—could exhibit similar responsiveness under their respective conditions. Yet, the extent and practical manifestation of this effect are governed by the intrinsic p*K*_a_ of each functional group, rendering the electrochemical window for ion regulation highly dependent on both molecular design and environmental context and not universally accessible within biologically relevant conditions. The intrinsic acidity of carboxyl groups ensures responsiveness within practical pH windows, demonstrating that fine-tuning molecular acidity is a critical prerequisite for precisely transcending and controlling ion dynamics and mixed conduction for advanced bioelectronic applications.

Despite achieving volumetric stability at pH ∼ 3.5, force measurements reveal substantial micromechanical changes during electrochemical doping (P3CBT and P3CPT-P, Figure S16). A pronounced increase in elastic modulus occurs upon doping, indicating that the polymer backbone and side chains experience localized mechanical changes even in the absence of measurable swelling. This contrast between macroscale stability and microscale mechanical response highlights a distinct mesoscale regime in which charge redistribution and ion compensation drive significant internal reorganization without large-scale dimensional expansion. These findings suggest that quasi-nonswelling behavior does not equate to mechanical passivity but rather reflects a finely balanced state where internal electrostatic forces and local conformational rearrangements are accommodated within geometrically constrained polymer matrices. Overall, this behavior confers a key advantage in precisely controlling swelling, positioning carboxylated OMIECs as promising candidates for mechanically robust devices within a biologically relevant pH range.

## 3 Conclusion and Outlook

Using a suite of *operando* multimodal characterization techniques, we reveal a pH-modulated doping mechanism governing charge compensation in carboxylated mixed conductors. The observed pH-dependent ion distributions originate from the intrinsic side chain chemistry, whose tunable dissociation–association equilibrium dictates overall ion dynamics. This coupling between chemical speciation and electrochemical behavior manifests across multiple length scales—from mesoscale (de)­swelling to nanoscale structural rearrangements—coherently modulating the material’s electrochemical, optoelectronic, and mechanical responses. By probing these dynamics at finer resolution, we identify a quasi-nonswelling regime near the critical pH, where electrochemical doping proceeds with nearly no volumetric change. This behavior underpins improved structural reversibility and suggests pathways toward enhanced long-term stability and biointerface compatibility, paving the way for realistic implantable and soft electronic applications.

While PCET typically governs n-channel OMIECs—where protons act as mobile compensating species—the mechanism in carboxylated p-channel polymers is distinct. Here, protons serve not as charge carriers but as chemical modulators of the fixed charge landscape, tuning side-chain deprotonation and thereby indirectly regulating mixed conduction. This decoupling of proton transport from electrochemical doping enables controlled, environment-responsive operation and unveils a unified framework for ionic–electronic–mechanical coupling in soft semiconductors. The ability to regulate ionic and volumetric responses through molecular acidity offers routes to electrochemical actuators with reversible deformation, while the sensitivity of structural and electronic states to pH and doping provides opportunities for light-responsive or photochemically coupled systems. Together, these insights establish a molecular-acidity-driven design paradigm for organic mixed conductors that seamlessly integrates electronic, mechanical, and photonic functionality for next-generation adaptive materials.

## 4 Methods

### 4.1 Polymer Solution and Film Preparation

P3K­(Pe)­T (poly­(3-potassium-5-pentanoate)­thiophene-2,5-diyl), Mw = 28 kDa, PDI = 2.0, Batch # BLS26-51); P3K­(He)­T (poly­(3-potassium-6-hexanoate)­thiophene-2,5-diyl), Mw = 22 kDa, PDI = 1.8, Batch # PTL34-75); P3K­(Hp)­T (poly­(3-potassium-7-heptanoate)­thiophene-2,5-diyl), Mw = 65 kDa, PDI = 2.6, Batch # BLS25-60); P3CBT (poly­(3-(4-carboxylbutyl)­thiophene-2,5-diyl), Mw = 39 kDa, PDI = 2.3, Batch # PTL39-74); P3CPT (poly­(3-(4-carboxylpentyl)­thiophene-2,5-diyl), Mw = 22 kDa, PDI = 1.8, Batch # BLS26-41); P3CHT (poly­(3-(4-carboxylhexyl)­thiophene-2,5-diyl), Mw = 65 kDa, PDI = 2.6, Batch # PTL39-70); P3EPT (poly­(3-(ethyl-5-pentanoate)­thiophene-2,5-diyl), Mw = 39 kDa, PDI = 2.3, Batch # PTL37-60); P3HT (poly­(3-hexylthiophene-2,5-diyl), Mw = 74 kDa, PDI = 2.2, Batch # PTL39-03); and P3MEEET (poly­(3-[2-[2-(2-methoxyethoxy)­ethoxy]­ethyl]­thiophene-2,5-diyl), Mw = 18 kDa, PDI = 1.6, Batch # BLS26-79) were purchased from Rieke Metals Inc. *p*-Toluenesulfonic acid monohydrate (pTsOH-H2O, 98%, Sigma-Aldrich), potassium chloride (99.9%, Sigma-Aldrich), potassium hexafluorophosphate (99.9%, Sigma-Aldrich), sodium hexafluorophosphate (99.9%, Sigma-Aldrich), dimethyl sulfoxide (99.9%, Sigma-Aldrich), 1,2-dichlorobenzene (99%, Sigma-Aldrich), acetone (99.5%, Sigma-Aldrich), anhydrous chloroform (99.9%, Sigma-Aldrich), methanol (99.8%, Sigma-Aldrich), and isopropyl alcohol (99.5%, Sigma-Aldrich) were used as received. Deionized water with a resistivity of 18.2 mΩ cm was obtained using an Aries water purification system. All protonated films, including P3CBT-P, P3CPT-P, and P3CHT-P, were fabricated following previously reported acidification methods^28,29,45^ by dissolving carboxylate salt into water at a concentration of 2 mg mL^–1^ followed by deposition and acidification to COOH. P3CBT, P3CPT, and P3CHT (2 mg) were dissolved in DMSO (1 mL); P3EPT (2 mg) was dissolved in 1,2-dichlorobenzene (1 mL); and P3MEEET (2 mg) was dissolved in chloroform (1 mL). All polymer solutions were stirred at room temperature overnight. They were then spray-cast using a gravity feed Iwata Eclipse HP-CS airbrush and heated to 60 °C for P3MEEET, 80 °C for P3CBT-P, and 130 °C for P3CBT and P3EPT. P3HT was dissolved in chloroform at 55 °C under stirring for 30 min, then left to cool to room temperature, and then spin-coated at a speed of 1500 rpm for 60 s. The films were cast onto various substrates, including glass, indium tin oxide (ITO)-coated glass (Delta Technologies, resistivity = 8–12 Ω sq^–1^) for *in situ* measurements, gold sensors for quartz crystal microbalance with dissipation (QCM-D) (Quartz PRO, QCM sensor, active area of 1.13 cm^2^), and silicon for grazing incidence wide-angle X-ray scattering (GIWAXS) characterization.

### 4.2 Contact Angle and Thickness Measurement

Contact angle measurements were performed on a ramé-hart Model 260 Standard Contact Angle Goniometer/Tensiometer with DI water and electrolyte on polymer-coated glass. Thickness measurements were performed using a 3D optical profilometer/interferometer system, ZeGage Pro.

### 4.3 Cyclic Voltammetry (CV)

CV (scan rate of 50 mV s^–1^ with a step size of 2 mV) was conducted using an AMETEK PMC 200 Potentiostat/Galvanostat and a three-electrode setup. The working electrode (WE) was prepared by spray-coating the polymer films onto ITO-coated glass slides (Delta Technologies, resistivity = 8–12 Ω sq^–1^). A platinum counter wire was used as the counter electrode (CE), and a standard Ag/AgCl electrode (3 M aqueous KCl inner solution, BASi) was used as the reference electrode (RE). 0.1 M KCl (*aq*) and K/NaPF_6_ (*aq*) solution was used as the electrolyte. To ensure accurate measurements, all electrolytes were degassed under argon flow (15 min) prior to and during the measurement process. pH values were accurately measured using an InLab Micro Pro-ISM pH sensor (Mettler-Toledo) interfaced with a Seven2Go pH/ion meter (Mettler-Toledo).

### 4.4 Quartz Crystal Microbalance with Dissipation Monitoring (QCM-D)

Passive swelling measurements were conducted with a Q-Sense Explorer Analyzer. First, the response of the bare Ti/Au sensors was recorded using QCM-D in air conditions, followed by measurements after the injection of 0.1 M KCl (*aq*) electrolyte into the chamber. These control measurements resulted in significant shifts in frequency and dissipation due to density differences between the media, which were excluded from the swelling percentage calculation. The sensors were then removed, and the conjugated polymer films were spray-coated directly onto the same sensors for P3CBT and P3EPT; additional acidification steps were performed to make the P3CBT-P thin film as previously described.^28,29,45^ The absolute difference in frequency between the bare sensor and the Ti/Au/polymer-coated sensors was compared using the “stitched data” function of Q-Soft software. This function accounted for density differences and allowed for the direct determination of mass changes per unit area by using the Sauerbrey equation (eq 1). The calculated mass changes were then converted to thickness changes, considering the sensor area and assuming a density of 1 g/cm^–3^ for the polymers in different states (dry and wet).

1 $$\frac{\Delta m}{A}=\frac{-17.7}{n}\Delta f_{n}$$

### 4.5 Electrochemical Quartz Crystal Microbalance with Dissipation Monitoring (EQCM-D)

Active swelling measurements under electrochemical doping/dedoping were performed using a Gamry interface 1010B coupled with a Q-Sense electrochemistry module (QEM401, Biolin Scientific). The three-electrode setup comprised an Ag/AgCl RE, a Pt CE, and a polymer-coated gold sensor as WE. All polymers were equilibrated by five cyclic voltammetry cycles from −0.3 to 0.6 V at a scan rate of 20 mV s^–1^. Frequency data and dissipation shifts were collected on the fifth, seventh, and ninth overtones for all polymers on QSotf401, and the data were further analyzed and fitted by D-find software.

### 4.6 Coarse-Grained Molecular Dynamics (CGMD) Simulations

The CG model of Savoie and coworkers for mixed ion-electron conductors in aqueous solutions^46,47^ was modified to represent the P3CBT polymer. The Savoie model is based on the Martini CG force field^48^ and incorporates an ellipsoidal CG bead to represent the planar thiophene ring. They demonstrated that their model mimics key experimentally observed trends in OMIEC materials,^46^ and in particular reproduces the bulk density and persistence length of P3HT.^47^ In this work, we modified the Savoie P3HT model by changing the outermost bead of the side chain to a Martini P3 bead type to mimic the polar COOH functionality of P3CBT. Simulations of a single polymer comprised of 20 P3CBT monomers were performed using the LAMMPS software.^49^ The polymer chain was dissolved in an aqueous solution with explicit Cl^–^ and K^+^ ions added to mimic a 0.1 M salt concentration. Additional Cl^–^ ions were added to neutralize the degree of backbone charge, which ranged from *q* = 0.05 to 0.2 per monomer to mimic different oxidation states along the doping process. To simulate the high pH deprotonated system, the end groups of the side chains were changed to Martini bead type Qa, which carries a negative charge. The appropriate number of K^+^ ions was added in the deprotonated case to maintain neutrality. Full details of the simulation methods are given in the SI.

### 4.7 Polymer Microstructural Analysis

Grazing incidence wide-angle X-ray scattering (GIWAXS) was performed at the Stanford Synchrotron Radiation Lightsource (SSRL) beamline 11-3, using an area detector (Rayonix MAR-225) with an incident energy of 12.7 keV. The sample-to-detector distance was 315.9 mm, calibrated using a LaB_6_ polycrystalline standard. The incidence angle was set to 0.12°, slightly above the critical angle, to sample the full film depth. All X-ray measurements were conducted in a He chamber to minimize air scattering and prevent beam damage to the samples. Raw data were normalized by detector counts and analyzed using the custom Python code.

### 4.8 *Operando* X-ray Scattering and Fluorescence

Grazing incidence X-ray fluorescence (GIXRF) and grazing incidence wide-angle X-ray scattering (GIWAXS) were performed simultaneously using a Frit cell at the SSRL beamline 17-2. The incident X-ray beam was focused to a size of ∼20 μm (vertical) × ∼100 μm (horizontal) with an incident angle of 0.12° and an energy of 11.6 keV. Fluorescence collection was performed using a Vortex silicon drift detector (SDD) positioned ∼70 mm from the sample. The detector was set to collect 9° above the sample plane and normal to the incident beam to avoid interference from the WE (pogo pin). The vertical and horizontal movement of the frit cell was motor-controlled, with a horizontal rocking motion of ± 0.4 mm applied during data collection to minimize beam damage to the film. The frit cell was housed in a hydrated, helium-purged environment. The potential control during the *operando* measurement was carried out using a PalmSens4 potentiostat with an Ag/AgCl electrode serving as the reference and counter electrode and a scan rate of 10 mV s^–1^. A stainless steel frit with a 20 μm average pore size was mounted on a sample block to facilitate electrolyte penetration. A silicon frit (MakroPor, thickness 350 μm, pore diameter 8 μm, and pore size 12 μm) was purchased from MilliporeSigma. A 5 nm Ti adhesion layer and a 50 nm Au layer were then deposited on the frit surface via vapor deposition. The polymer was subsequently float-transferred onto the top of the frit, serving as the working electrode.

### 4.9 *Operando* Raman Spectroscopy

Raman spectra were obtained using a Horiba LabRAM Odyssey confocal Raman microscope with a 633 nm excitation laser source in backscattering geometry. The spectra of the dry films were measured through a 50× objective, while the spectra of films exposed to the electrolyte were measured using a 5× objective. The laser power was set at 50% (8.5 mW) for all conditions to avoid photothermal effects and sample degradation. For all measurements, thin films of target polymers were spray cast onto ITO/glass substrates (8–12 Ω sq^–1^ Delta Technologies), then acidified as per above, and used as working electrodes. An Ag/AgCl pellet (*D* = 2 mm × *H* = 2 mm, Warner Instruments) and Pt wire were used as the RE and CE, respectively. A Gamry Interface 1000 Potentiostat was used to perform *in situ* doping and dedoping experiments. All measurements were taken in an AIST-NT EC001 electrochemical cell filled with 0.1 M NaCl (aq) electrolyte. The spectral region scanned in this investigation ranged between 600 and 1799 cm^–1^. A silicon wafer was used for the calibration process, and all spectra were collected using LabSpec 6 software and further deconvoluted through PeakFit version 4.12 software.

### 4.10 *Operando* Transient Absorption Spectroscopy

*Operando* transient absorption spectroscopy (TAS) was performed with an Ultrafast Systems Helios spectrometer. One hundred fifty femtosecond (fs) pulses of 800 nm laser light were generated with a Coherent Libra amplified Ti:sapphire system at 1.2 W and 1 kHz repetition rate. Experiments were conducted using a 850 nm pump that was attenuated to 1.0 mW and a probe generated using a sapphire crystal, yielding an optical window of 800 nm–1600 nm. The transient absorption spectra were measured over a 200 ps window. For each scan, 100 time points were recorded with exponential spacing, and each sample was subjected to three scans that were averaged to create the complete data set. A reference point was used to align the reflectance of the sample in the holder to maintain consistency in the angle of incidence for the pump beam for each experiment. Select scans for each sample were compared before analysis to ensure that no degradation of signal had occurred during the experiment and to ensure data consistency. The TAS signal intensity is typically presented as a change in absorption (ΔA), which can broadly be understood as the difference between the excited-state and ground-state absorption. Data preparation, including chirp correction, was performed using the global analysis software Surface Xplorer, provided free of charge by Ultrafast Systems. A Gamry Interface 1000 Potentiostat was used to perform *in situ* doping and dedoping experiments. All measurements were taken in quartz cuvettes and filled with electrolyte.

### 4.11 *Operando* Atomic Force Microscopy (AFM) and Force Measurements

AFM measurements were performed *in situ* in the electrolytes listed using an Oxford Instruments/Asylum Research Cypher-ES. All cantilevers were calibrated on a sapphire reference sample in the same electrolyte to correct for spring constant and optical lever sensitivity, using a gold-coated contact mode cantilever (BudgetSensors ContGB-G, *k* ∼ 0.2 N/m, ambient *f*_0_ ∼13 kHz) for force mapping and Cr/Pt-coated cantilevers (BudgetSensors, Multi75E-G, *k* ∼ 2 N/m, ambient *f*_0_ ∼ 75 kHz). For height measurements, data were taken in AC mode using a razor blade scratch on ITO as a reference. The AFM chamber was purged with nitrogen prior to being sealed. The bias voltage in all data was applied to the cantilever. Data were analyzed by using Igor Pro.

### 4.12 *Operando* Ultraviolet–Visible–Near-Infrared (UV–Vis–NIR) Spectroelectrochemistry

Measurements were performed using the same setup as the CV experiment, with a three-electrode configuration in an aqueous solution under a nitrogen atmosphere. The WE and RE were polymer-coated ITO and Ag/AgCl, respectively, consistent with the CV experiment described above, with a platinum wire (Gamry) used as the CE. The experiment was conducted using an Agilent Cary 5000 spectrophotometer with quartz cuvettes having a path length of 1 cm. The polymer thin film was biased under potentiostatic conditions, controlled by a Gamry Interface 1010B potentiostat. The film spectra were recorded once the WE current reached a steady state, which typically required 30 to 60 s for each potential step.

### 4.13 OECT Fabrication and Characterization

Interdigitated organic electrochemical transistor (iOECT) substrates were purchased from MicruX Technologies (Model ED-IDE1-Au, total area = 38.5 mm^2^, 10 μm electrode length, 10 μm electrode gap, gold thickness = 150 nm, active area = 9.6 mm^2^, average width = 2.75 mm), and devices were fabricated using prior published methods.^28^ A cylinder-shaped Ag/AgCl pellet (Warner Instruments) was used as a gate electrode and immersed in a 0.1 M aqueous NaCl solution confined in a PDMS well. Transfer and output characteristics were measured with an Agilent B1500A semiconductor analyzer. All transistor characteristics were collected using Keysight Easy Expert software with a custom *I*/*V* sweep configuration and were tested under ambient conditions. Data were collected for at least five separate devices to ensure reproducibility. Output curves were collected with a *V*_d_ step size of −0.1 mV and a *V*_g_ step size of – 0.1 V. Transfer curves were collected using a *V*_d_ = −0.6 V with a *V*_g_ step size of −0.1 mV.

## Supplementary Material



## Notes

Cyclic voltammetry (CV), passive swelling measurement, contact angle measurements, discussion for coarse-grained model (CG) and molecular dynamics (MD), *operando* grazing incidence X-ray fluorescence (GIXRF), *in situ* atomic force microscopy (AFM), *ex situ* grazing incidence wide-angle X-ray scattering (GIWAXS), electrochemical quartz crystal microbalance with dissipation energy (EQCM-D), *operando* spectroelectrochemistry, *operando* transient absorption spectroscopy (TAS), discussion for quantification and identification of ionic species across pH, and *operando* force mapping (PDF)
