# Supplementary material for: pH Regulates Ion Dynamics in Carboxylated Mixed Conductors
Source: Chem Mater. 2026 Feb 9;38(4):2041–54. doi: 10.1021/acs.chemmater.5c03288 (PMC12937051; doi:10.1021/acs.chemmater.5c03288)
Supplement: Supplementary file 1 [file cm5c03288_si_001.pdf]

Supporting information:

pH Regulates Ion Dynamics in Carboxylated Mixed Conductors

Zeyuan Sun,[1] Mengting Sun,[1] Rajiv Giridharagopal,[2] Robert C. Hamburger,[3] Siyu Qin,[1] Haoxuan Li,[1] Mitchell Hausback,[1] Yulong Zheng,[4] Bohyeon Kim,[1] Heng Tan,[5] Thomas E. Gartner III,[1*] Elizabeth R. Young,[3*] Christopher J Takacs,[6*] David S. Ginger,[2*] Elsa Reichmanis[1*]

[1]Department of Chemical and Biomolecular Engineering, Lehigh University, Bethlehem, PA 18015, United States

[2]Department of Chemistry, University of Washington, Seattle, WA 98195, United States

[3]Department of Chemistry, Lehigh University, Bethlehem, PA 18015, United States

[4]School of Chemistry and Biochemistry, Georgia Institute of Technology, Atlanta, GA 30332, United States

[5]Department of Computer Science and Engineering, Lehigh University, Bethlehem, PA 18015, United States

[6]Stanford Synchrotron Radiation Lightsource SLAC National Accelerator Laboratory, Menlo Park, CA 94025, United States

Table of Contents

Supplementary Figures

Figure S1: *Cation-independent behavior and Concentration calculation based on pH titration methods*

Figure S2: *Concentration-independent behavior*

Figure S3: *Experimental hydrophilicity study*

Figure S4: *Contact angle measurement*

Figure S5: *Operando GIXRF raw data during doping and dedoping*

Figure S6: *Elastic scattering peak from the operando GIXRF data in Fig. S6*

Figure S7: *Absolute thickness changes in in-situ AFM for P3CBT-P under neutral and acidic pH conditions.*

Figure S8: *2D GIWAXS patterns of carboxylated polythiophene series.*

Figure S9: *2D line cut profiles for all the series respectively.*

Figure S10: *Side-by-side comparison of P3CHT (DMSO) under different pH conditions*

Figure S11: *Operando spectroelectrochemistry and pre / self doped evidence.*

Figure S12: *TAS raw data for carboxylated polythiophene under pH neutral condition.*

Figure S13: *TAS raw data for carboxylated polythiophene under pH acidic condition.*

Figure S14: *Kinetic trace at λ_{obs} = 1000 nm for TAS measurements on P3CBT-P under different conditions.*

Figure S15: *Open-circuit potential (OCP or V_{oc}) regulates ion uptakes in carboxylated mixed conductors.*

Figure S16: *Operando force mapping*

Supplementary Discussions

Discussion 1: *Coarse-Grained Model / Parameterization / Molecular Dynamics*

Discussion 2: *Quantify fixed-charge density vs pH and identify dominating ionic species*

Supplementary Tables

Table S1: *Bonded interaction parameters. Units of all parameters are consistent with LAMMPS "real" units (kcal/mol for k, Å for r). Angle units are in degrees.*

Table S2: *MARTINI bead assignments*

Table S3: *Non-bonded interaction parameters. In LAMMPS "real" units (kcal/mol for ε, Å for σ).*

Table S4: *Qualitative interpretation of Open-Circuit Potentials in determining preference in OCP values for P3CBT-P and P3CBT in pH varied KCl electrolyte.*

References

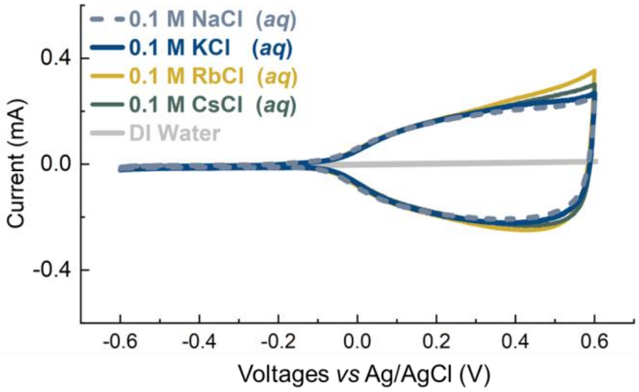

Figure S1: **Cation-independent behavior**. Cyclic voltammetry (CV) was performed on P3CBT-P in chloride-based aqueous electrolytes containing different monovalent cations, under conditions of neutral pH and comparable thickness. The voltammogram reveals a largely cation-independent electrochemical response, with sodium and potassium electrolyte exhibiting negligible differences in performance – supporting their use as valid reference for PF_6 -based system. Slight increases in peak current were observed with rubidium and cesium chlorides, but all chloride salts showed similar turn-on voltage. In contrast measurements in deionized water, used as a background control, showed no detectable conductivity.

<u>Concentration calculation based on pH titration methods:</u>

The pH of 100 ml of 0.1 M KCl electrolyte is regulated through titrating concentrated HCl solution, where the pH is related to the concentration of protons by the equation:

Molarity of concentrated HCl, where the 37% w/w HCl solution, with 37 g per 100 g solution, with a density of 1.19 g/ml, and a molar mass of 36.46 g/mol

Therefore, the molarity of HCl is:

$$HCl = \frac{37g \div 36.46g/mol}{100\ g\ \div 1.19\ g/ml} = \frac{1.015\ mol}{0.08403\ L} = 12.08\ M$$

Moles of HCl required to reach pH 2.5:

$$[H^+] = 10^{-2.5} = 3.16 \times 10^{-3} mol/L$$

To reach this proton concentration in ~100 ml of solution:

$$mol\ HCl\ needed = 3.16\ \times 10^{-3} mol/L\ \times 0.1\ L = 3.16 \times 10^{-4} mol$$

$$Volume\ of\ 12.08\ M\ HCl\ added = \frac{3.16\ \times 10^{-4} mol}{12.08\ mol/L} = 26.2\ \mu L$$

Total moles of Cl^- in the final solution:

$$[Cl^-] = \frac{0.010\ mol + 0.000316\ mol}{100\ ml + 26.2\ \mu L} = 0.1031\ M$$

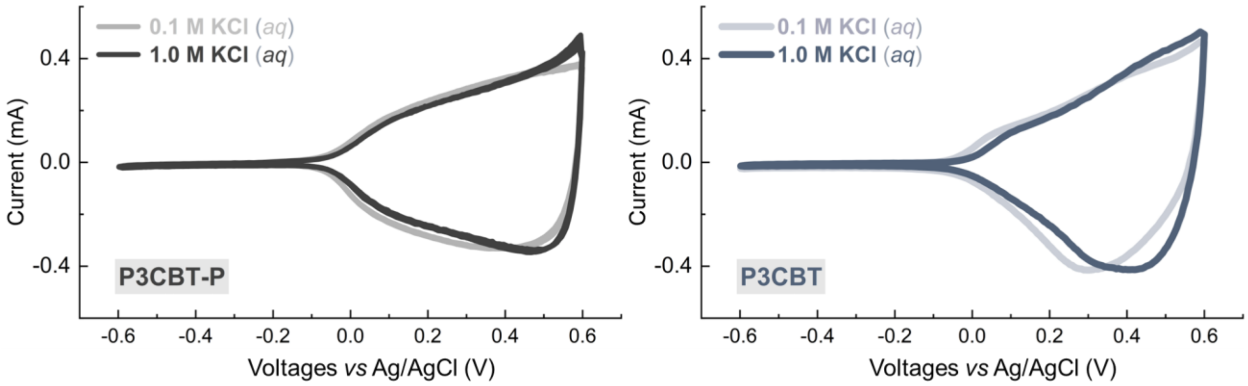

Figure S2: **Concentration-independent behavior**. Cyclic voltammetry (CV) was performed on P3CBT-P and P3CBT in chloride-based aqueous electrolytes of varying concentration (0.1 – 1 M), with neutral pH and comparable film across samples. The voltammogram show that changes in potassium or chloride ion within this range do not significantly affect the electrochemical response, indicating that ion concentration does not play a significant role in the pH-dependent behavior throughout this study, and the voltage offset shown in main figure 1 is mainly due to the pH induced dissociation change.

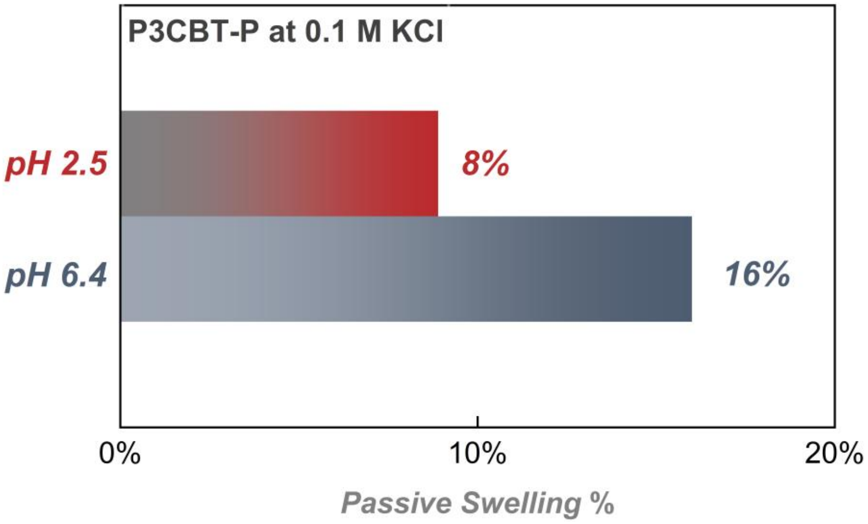

Figure S3: **Experimental hydrophilicity study**. Comparison of passive swelling percentage comparison for P3CBT-P in 0.1 M acidic KCl (red) and neutral KCl (blue). Combined with GIXRF data, this result indicates reduced cation uptake under acidic conditions. This suggests that passive swelling is primarily influenced by cation-polymer interactions and the intrinsic hydrophilicity (degree of dissociation) of the polymer.

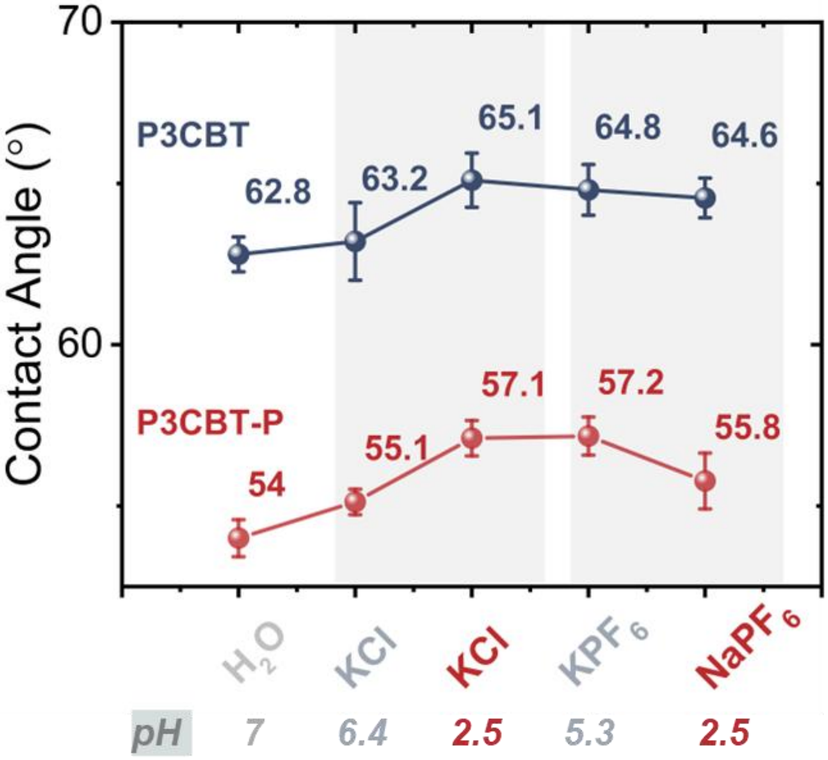

Figure S4: Contact angle measurement of thin films for P3CBT-P (red) P3CBT (blue), and P3EPT (light brown) average from five samples in H_2O, 0.1 M KCl at neutral pH, 0.1 M KCl at acidic pH, and 0.1 M KPF_6 at neutral pH and $NaPF_6$ at acidic pH

Discussion 1: Coarse-Grained Model

We created a model for P3CBT based on the model developed by Savoie and coworkers for P3HT.[1] The Savoie model is itself based on the MARTINI coarse-grained (CG) force field[2] and added ellipsoidal species to capture the anisotropic nature of thiophene rings.[3] In this work, we adjusted the P3HT model to represent P3CBT by changing the side chain species. The P3CBT system was represented using six main components: backbone, non-polar side chain, polar side chain, anion, cation, and solvent. Each polymer monomer was mapped to three CG beads: one anisotropic ellipsoidal backbone bead (BB), one spherical non-polar side chain bead (NS), and one spherical polar side chain bead (PS).

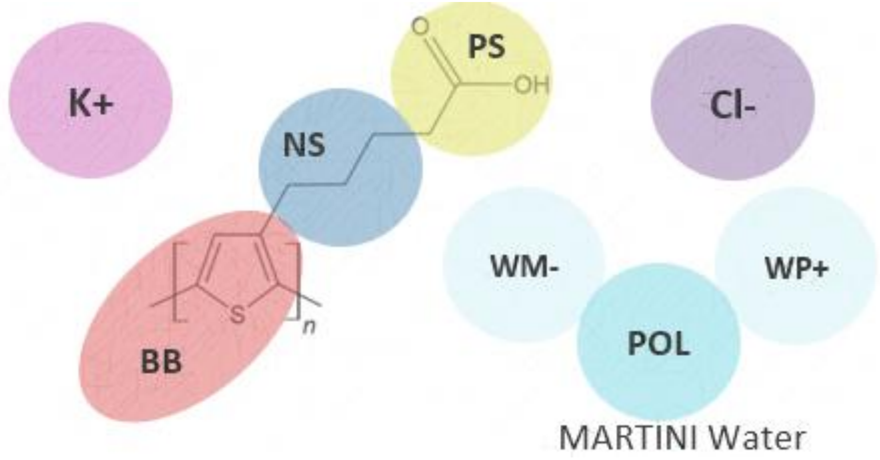

Figure shows Coarse-grained representation of the P3CBT system

Non-bonded interactions between spherical CG beads were computed using the Lennard-Jones (LJ) and Coulomb potentials with shifting cutoff functions $S_{LJ}(r)$ and $S_C(r)$ shown in **Equations 1 to 3**.[4] The LAMMPS pair style "lj/gromacs/coul/gromacs" was employed.[5]

$$U_{12}(r) = 4\varepsilon\left[\left(\frac{\sigma}{r}\right)^{12} - \left(\frac{\sigma}{r}\right)^{6}\right] + S_{LJ}(r) \tag{1}$$

$$U_{12}(r) = \frac{Cq_1q_2}{\epsilon r} + S_C(r) \tag{2}$$

Where U_{12} is the potential between particles 1 and 2, r is the center-to-center distance between particles, σ is the distance at which the interaction potential equals zero, ε is the depth of the energy well, C is a coefficient computed by LAMMPS, and q_1, q_2 are the charges of particles 1 and 2. The LJ potential used an inner cut-off of 9 Å and an outer cut-off of 12 Å. For the Coulomb potential, only an outer cut-off of 12 Å was applied, with the inner cut-off set to 0 Å.

Backbone-backbone interactions between ellipsoidal beads were treated with the Gay-Berne (GB) potential to capture the anisotropic interactions with semi-axis lengths σ_x, σ_y, and σ_z.

$$U_{12}(\omega_1,\omega_2,r_{12}) = 4\varepsilon_0\varepsilon_{12}^{v}(\omega_1,\omega_2)\varepsilon_{12}'^{\mu}(\omega_1,\omega_2,\hat{r}_{12})$$

$$\times\left\{\left[\frac{\sigma_c}{r_{12}-\sigma_{12}(\omega_1,\omega_2,\hat{r}_{12})+\sigma_c}\right]^{12} - \left[\frac{\sigma_c}{r_{12}-\sigma_{12}(\omega_1,\omega_2,\hat{r}_{12})+\sigma_c}\right]^{6}\right\} \tag{3}$$

ω_1 and ω_2 are the Euler angles of the particles, r_{12} is the displacement vector, ε_{12} and ε'_{12} are energy prefactors, σ_c is the minimum contact distance, and ε_0 is the well depth. The exponents v and μ are empirical coefficients that are set to one. A 12 Å cutoff was applied. Directly bonded neighbors within a given molecule were excluded from the nonbonded interaction calculation.

Bonded potentials involving backbone beads were computed using custom LAMMPS classes developed by the Savoie group[3]: "elel" (ellipsoid-ellipsoid, backbone-backbone interaction) and "elsp" (ellipsoid-sphere, backbone-side chain interaction). All other bonded interactions used standard LAMMPS potentials, namely harmonic bonds, cosine-squared angles, and OPLS dihedrals. The full set of bonded parameters is provided in **Table S1**.

Table S1: Bonded interaction parameters. Units of all parameters are consistent with LAMMPS "real" units (kcal/mol for k, Å for r). Angle units are in degrees.

Bonds		Angles	
BB-BB	$k = 25.0, r_0 = 4.3$	BB-BB-BB	$k_a = 1.0, \theta_0 = 160$
BB-NS	$k = 1.5, r_0 = 4.7$	BB-BB-NS	$k_a = 3.0, \theta_0 = 90$
NS-PS	$k = 1.5, r_0 = 4.6$	BB-NS-PS	$k_a = 3.0, \theta_0 = 180$
POL-WP, POL-WM	$k = 1.0, r_0 = 1.4$	WM-POL-WP	$k_a = 0.5019, \theta_0 = 0$
Dihedrals		Parameters for Custom Potentials	
BB-BB-BB-BB	$K_1 = 0.5, K_2 = 0.6$<br>$K_3 = 0.0, K_4 = 0.0$	BB-BB, d	$K_1 = 0.0, K_2 = 2.0$<br>$K_3 = 0.0, K_4 = 0.0$
NS-BB-BB-NS	$K_1 = 3.0, K_2 = 0.0$<br>$K_3 = 0.0, K_4 = 0.0$	BB-BB, a_1	$k_a = 7.5, r_0 = 90$
		BB-BB, a_2	$k_a = 7.5, r_0 = 90$
		BB-NS, a	$k_a = 3.0, r_0 = 90$

The size, mass, and charge of each coarse-grained bead were specified through the LAMMPS atom style. A custom atom style, *hybrid full ellipsoid*, was used to define ellipsoidal backbone beads, particle charges, and molecular connectivity. To enable these features, the MOLECULE, ASPHERE, and EXTRA-PAIR packages were required during LAMMPS compilation. The bead mass is not assigned directly in LAMMPS but instead calculated from bead volume and density shown in **Equation 4**:

$$Mass = Volume \times Density = \frac{4}{3}\pi R_x R_y R_z \times Density \tag{4}$$

Where R_x, R_y, and R_z are the bead raddi in the x, y, and z directions. The diameter and density of each bead are defined within the LAMMPS input script.

Parameterization

Nonbonded interactions were parameterized using the MARTINI CG force field, originally developed for lipid and biomolecular systems but now widely applied to conjugated polymers.[2] The MARTINI framework provides a transferable library of bead types calibrated against experimental partitioning free energies, while maintaining computational efficiency by grouping about four heavy atoms into a single coarse-grained site. This mapping preserves essential chemical properties such as

polarity, charge, and hydrogen-bonding capability, while allowing simulations to reach the larger system sizes and longer timescales. Within the MARTINI force field, bead types are categorized according to polarity: polar (P), nonpolar (N), apolar (C), or charged (Q). Each category is further refined by numerical indices (1–5, from least to most polar) or hydrogen-bonding properties: "a" for acceptor, "d" for donor, "da" for both, and "0" for none. In addition, the force field defines a set of "small" beads, designated with the prefix *S*, which represent a reduced effective volume compared to standard beads and are often used to describe sterically restricted groups such as short sidechains. The MARTINI bead assignments for each component in our system are listed in **Table S2**.

Table S2: MARTINI bead assignments

Component	MARTINI Bead
Backbone (BB)	C4
Non-polar Sidechain (NS)	SC3
Polar Sidechain (PS)	P3
Anion (Q)	Qa
Cation (Q)	Q0

Each thiophene unit in P3CBT was represented as an ellipsoidal bead. The in-plane dimensions of the ellipsoid (x and y) were set to 5 Å, corresponding to the approximate diameter of a thiophene ring, while the out-of-plane dimension (z) was set to 3 Å, consistent with the average π–π stacking distance.[6,7] This anisotropic representation allows the coarse-grained model to reproduce direction-dependent interactions critical for backbone ordering. All other components of the system were modeled as finite-sized spherical beads. For spherical beads, the diameter was chosen to match the self-interaction zero-energy distance (σ) from the LJ potential model. In this system, spherical beads were assigned to a diameter of 4.7 Å, except for the non-polar sidechain bead, which was slightly smaller at 4.3 Å.

Cations and anions in the model served both as explicit KCl salt and as compensating charges for the polymer. Potassium ions (K^+) were assigned a charge of +1e, and chloride ions (Cl^-) a charge of −1e. Additional anionic or cationic beads (−1e or +1e) were introduced as needed to maintain charge neutrality, either balancing the positively charged backbone or the negatively charged polar sidechains in deprotonated systems. Consistent with previous coarse-grained models of OMIECs, the oxidative charge on the polymer backbone was assumed to be uniformly distributed across all monomers.[8]

The solvent was represented using the MARTINI polarizable water model.[9] In this model, three coarse-grained beads correspond to four real water molecules, enabling explicit treatment of orientational polarizability. The WP and WM beads carry charges of +0.46e and –0.46e, respectively,

and a global dielectric constant of 2.5 was applied. This water representation allows for a more realistic description of hydration and ion solvation compared to nonpolarizable models. For MARTINI polarizable water, intramolecular WP/WM interactions were excluded to preserve rotational polarizability. The parameters for all non-bonded interactions, including solvent, are provided in **Table S3**.

Table S3: Non-bonded interaction parameters. In LAMMPS "real" units (kcal/mol for ε, Å for σ).

	BB	NS	PS	POL	Qa	Q0
BB	$\sigma_x = \sigma_y = 5.0$, $\sigma_z = 3.0$ $\varepsilon_x = \varepsilon_y = 0.25$, $\varepsilon_z = 1.2$ $\varepsilon_0 = 1.0, \sigma_c = 3.0$	$\varepsilon = 0.84$ $\sigma = 4.7$	$\varepsilon = 0.74$ $\sigma = 4.7$	$\varepsilon = 0.61$ $\sigma = 4.7$	$\varepsilon = 0.74$ $\sigma = 4.7$	$\varepsilon = 0.74$ $\sigma = 4.7$
NS		$\varepsilon = 0.63$ $\sigma = 4.3$	$\varepsilon = 0.74$ $\sigma = 4.7$	$\varepsilon = 0.61$ $\sigma = 4.7$	$\varepsilon = 0.65$ $\sigma = 4.7$	$\varepsilon = 0.65$ $\sigma = 4.7$
PS			$\varepsilon = 1.19$ $\sigma = 4.7$	$\varepsilon = 1.14$ $\sigma = 4.7$	$\varepsilon = 1.34$ $\sigma = 4.7$	$\varepsilon = 1.34$ $\sigma = 4.7$
POL				$\varepsilon = 0.96$ $\sigma = 4.7$	$\varepsilon = 1.19$ $\sigma = 4.7$	$\varepsilon = 1.07$ $\sigma = 4.7$
Qa					$\varepsilon = 0.84$ $\sigma = 4.7$	$\varepsilon = 0.55$ $\sigma = 4.7$
Q0						$\varepsilon = 0.84$ $\sigma = 4.7$

The effect of pH was modeled by varying the protonation state of the carboxylic acid end group on the sidechains. The only difference between the acidic and neutral conditions was the MARTINI bead representation of the carboxylic acid group. The Henderson-Hasselbalch Equation (**Equations 5 and 6**) was used to calculate the percentage of deprotonation for a given pH. Assuming a pKa of 4.5 for the carboxylic acid, experimental conditions at pH 2.5 and 6.4 correspond to calculated deprotonation percentages of ~1% and ~99%, respectively. Accordingly, the acidic condition (pH 2.5) was modeled as fully protonated, where the sidechain end group was represented by a neutral P3 bead. The neutral condition (pH 6.4) was modeled as fully deprotonated, where the sidechain end group was represented by a negatively charged Qa bead corresponding to COO^-.

$$pH = pKa + \log_{10}\left(\frac{[A^-]}{[HA]}\right) \tag{5}$$

$$Deprotonated\,\% = \frac{[A^-]}{[A^-] + [HA]} \times 100\% = \frac{10^{pH-pKa}}{10^{pH-pKa} + 1} \times 100\% \tag{6}$$

Molecular Dynamics

All molecular dynamics simulations were performed using LAMMPS[10] with a 10 fs timestep and Velocity–Verlet integration under periodic boundary conditions. Systems were initialized in a cubic simulation box with a 4050 Å box side length using Moltemplate,[11] which placed molecules at random locations chosen from a diffuse grid. A 150 Å lattice spacing along all three axes were used to ensure components were non-overlapping. The bead velocities are initialized using a randomly seeded uniform distribution to give a system temperature of 300K. The system was equilibrated through a multi-stage protocol. First, an NVE relaxation was performed for 10 ps with bead displacements limited to 0.1 Å per timestep. This was followed by a 1 ns NVT simulation in which the simulation box was linearly deformed until the system density reached 1.0 g/cm³. After this step, beads velocities were rescaled to 300 K using another randomly seeded uniform distribution to prevent the buildup of translational or rotational momentum. A second short NVE relaxation of 50 ps with displacement restraints was then carried out, after which the system was equilibrated in the NPT ensemble at 300 K and 1 atm for 5 ns. To stabilize the density, an additional NVT run of 5 ns was performed at 300K using the average density from the NPT step. Finally, production simulations were carried out in the NPT ensemble at 300 K for 50 ns. Trajectory data in the production run was recorded every 10,000 timesteps, corresponding to an interval of 100 ps. This resulted in 500 frames for radial distribution function, g(r) analysis. Throughout equilibration and production, the linear and angular momentum of the system were zeroed every 10 ps.

Each simulation contained 1 polymer chain of 20 monomers each, along with 65 K^+ cations and Cl^- anions, and 9000 MARTINI water molecules (corresponding to 36000 real water molecules). The ion concentration was chosen to represent a background electrolyte composition of 0.1 M. Additional ion content was adjusted according to backbone charge and protonation state to maintain overall system charge neutrality. Three independent replicates were performed for each of the four states considered in this work: two backbone charge states ($q = 0.05e$ and $q = 0.2e$) and two deprotonation states (fully protonated and fully deprotonated).

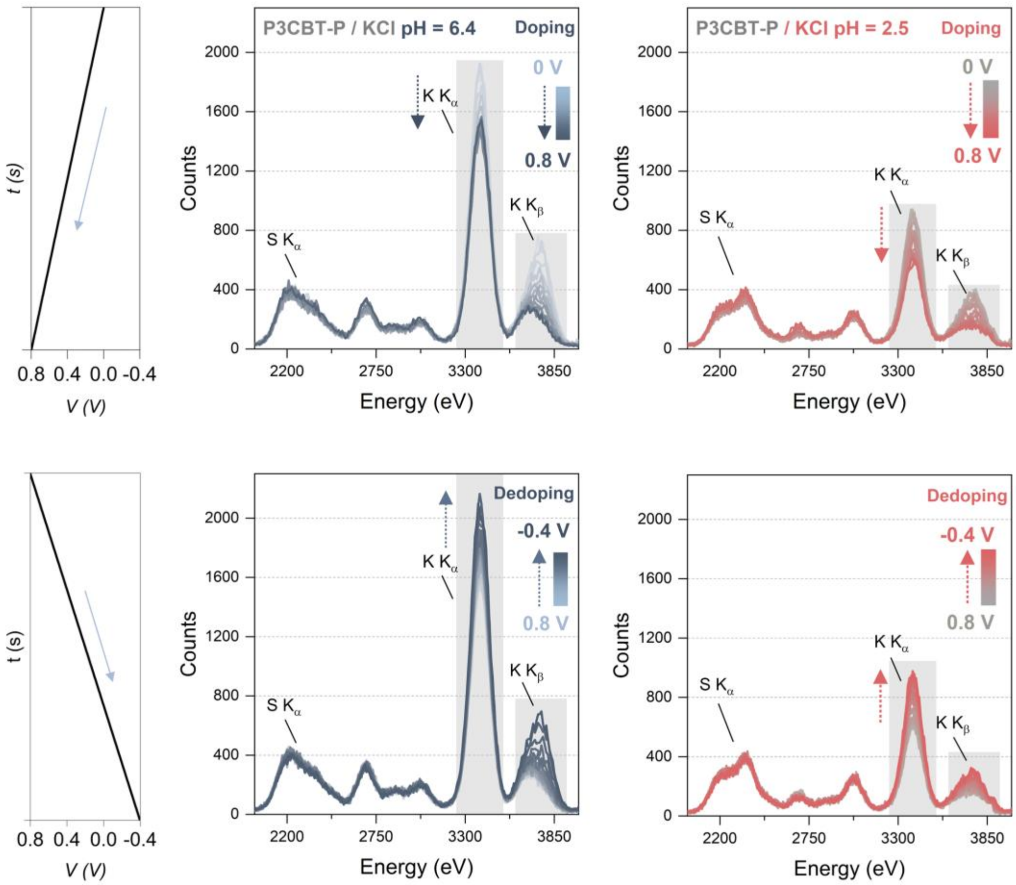

Figure S5: ***Operando* GIXRF during (de)doping**. Raw data for P3CBT-P in 0.1 M KCl at pH 6.4 (blue), and pH 2.5 (red), collected during doping (0 V to 0.8 V, top row), and dedoping (0.8 V to -0.4 V, bottom row) at a rate of 10 mV s^{-1}.

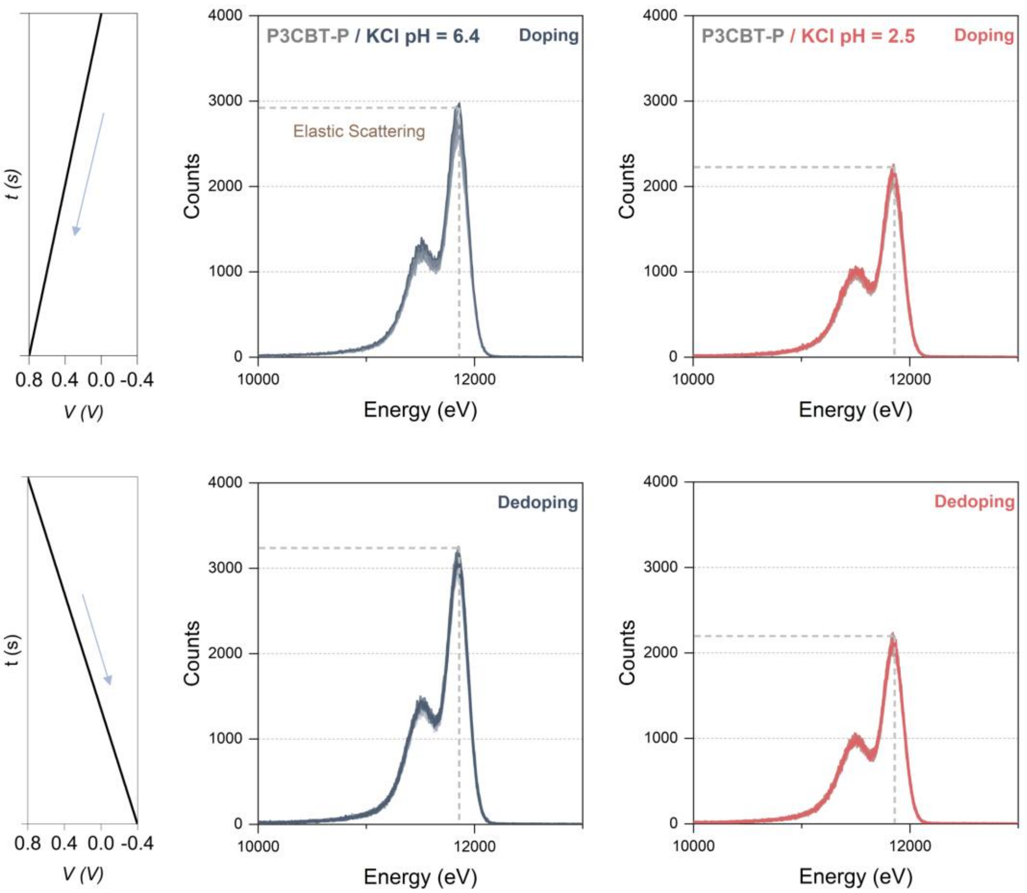

Figure S6: Elastic scattering peak from the *operando* GIXRF data in **Fig. S5**, used for beam energy calibration and normalization purpose to enable qualitative comparison.

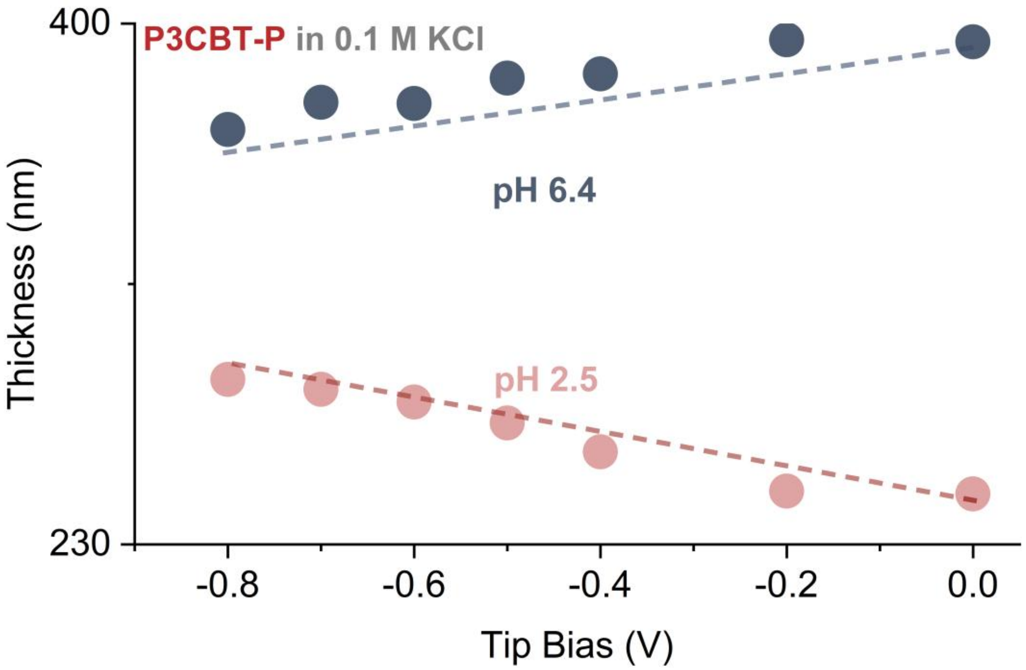

Figure S7: Absolute thickness changes in *in situ* AFM for P3CBT-P under neutral and acidic pH conditions.

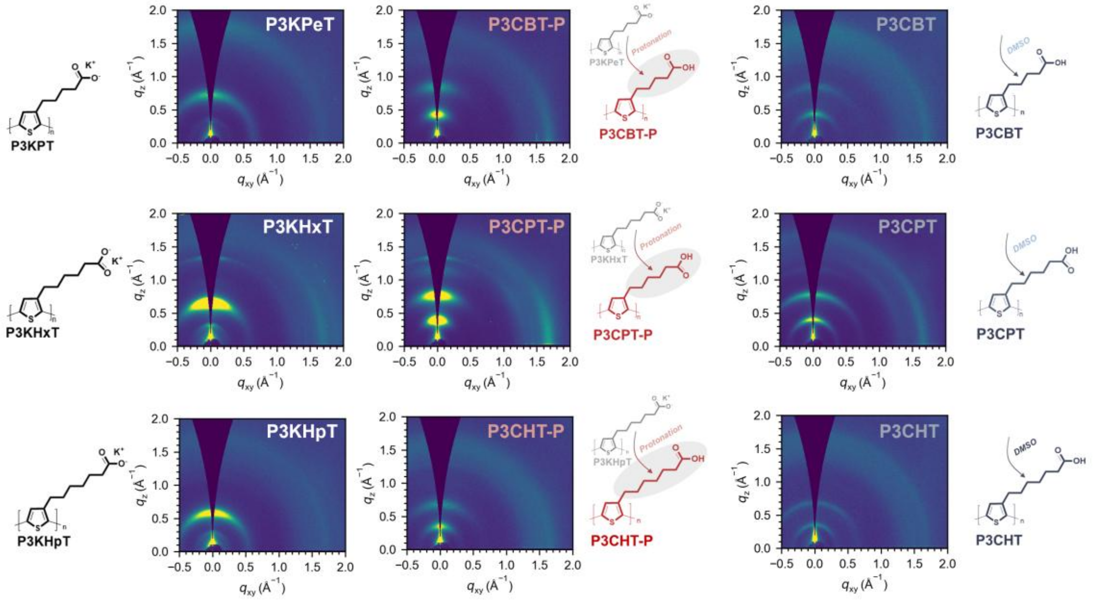

Figure S8: 2D GIWAXS patterns of carboxylated polythiophenes. The left column shows the **COOK** series (water soluble carboxylate form): P3KPeT, P3KHxT, and P3KHpT from top to bottom. The middle column presents the **protonated COOH** series (carboxylic acid form by acidification): P3CBT-P, P3CPT-P, and P3CHT-P. The right column displays the **pristine COOH** series (carboxylic acid in DMSO): P3CBT, P3CPT, and P3CHT from top to bottom. While molecular weight may influence the extend of deswelling/swelling quantitively, the qualitative trend observed should be consistent across the series studied here.

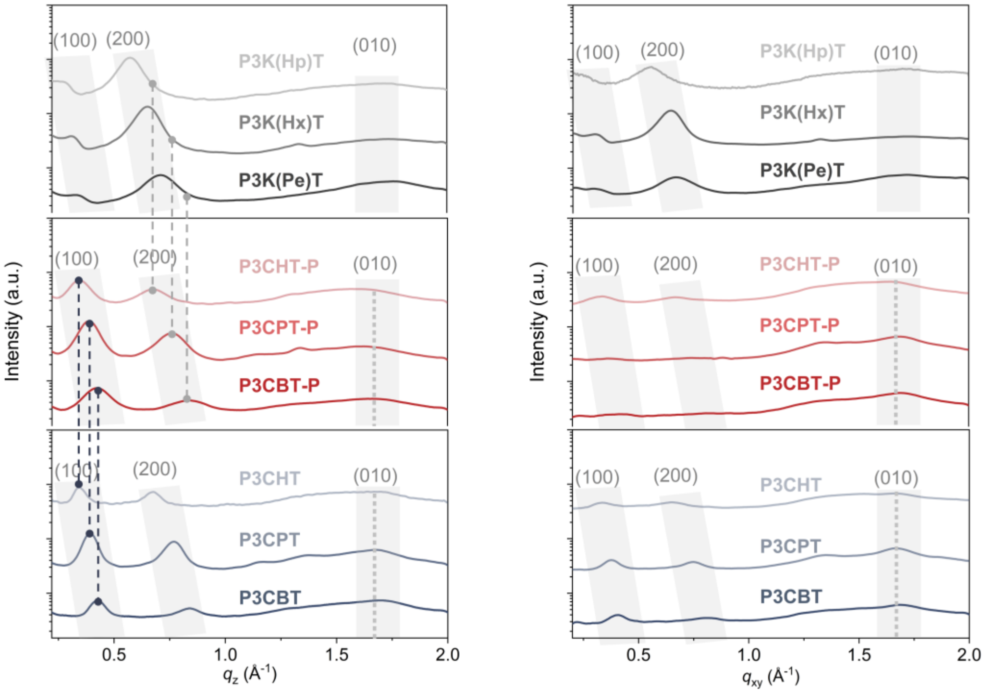

Figure S9: the corresponding 2D line cut profiles for all the series respectively. These Figure **S8** and **S9** results indicate that:

1) We observe that the peak intensity for (200) is more intense than (100) in carboxylate salk (COOK) samples, and disappear after protonation. This effect likely arises from changes in the lamellar electron-density distribution induced by potassium ions, which increase the local electron density and alter the symmetry of the lamellar electron-density profile. As a result, the scattering intensity is redistributed among different (h00) reflections. After protonation, removal of these ions restores a simpler lamellar electron-density distribution and the conventional dominance of the (100) reflection. This is consistent with previous observations in modified polythiophene system.[12,13] Similar results also show in a donor-acceptor conjugated polyelectrolyte CPE-K.[14]

2) Increasing the side chain spacer significantly expands the lamellar spacing, while π–π stacking remains largely unaffected across all polymers.

3) As previously shown,[15] the K-to-H conversion significantly influences molecular arrangement and packing. The resulting change in lamellar spacing is expected due to volumetric effects from ion exchange. With longer side chain spacers, the protonated COOH polymers become more isotropic, with P3CHT-P exhibiting the highest isotropy among the series. In contrast, the DMSO-treated COOH series displays consistently isotropic microstructures across all variants, differing from the protonated counterparts.

4) The peak positions remain unaffected by processing conditions, with all COOH polymers exhibiting identical lamellar and π–π stacking peaks. Microstructurally, the longest side-chain polymer serves as a representative for both series due to its structural similarity and comparable/beneficial crystalline features observed in X-ray scattering experiments.

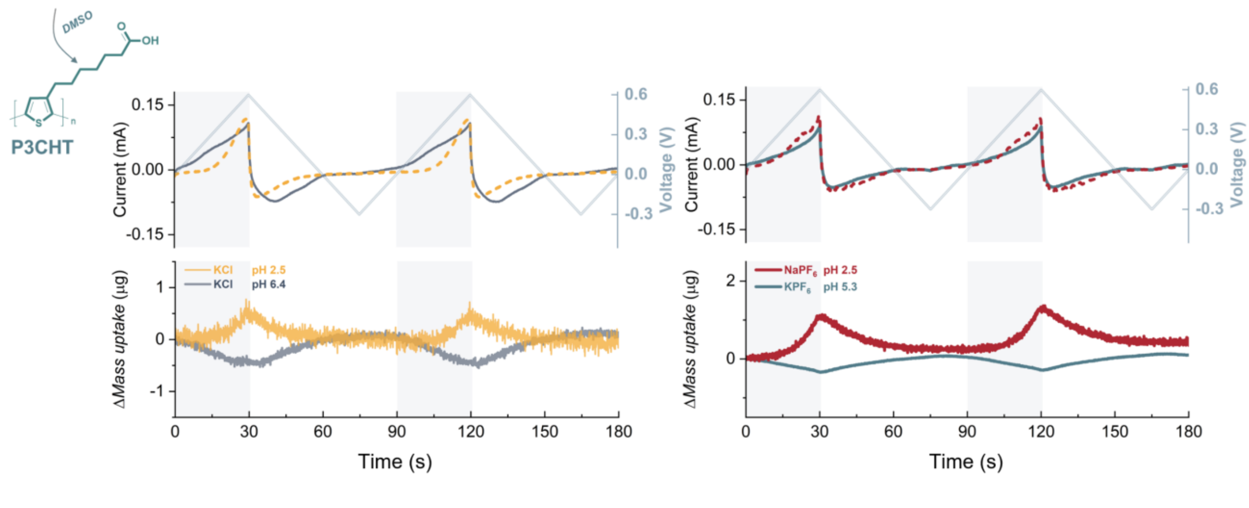

Figure. S10: Side-by-side comparison of P3CHT (DMSO) under different pH conditions in chloride-based (left) and PF_6^--based (right) electrolytes. Both cases exhibit clear pH-dependent swelling behavior, indicating that P3CHT can serve as a representative model for carboxyl-functionalized polythiophenes in GIWAXS and GIXRF studies

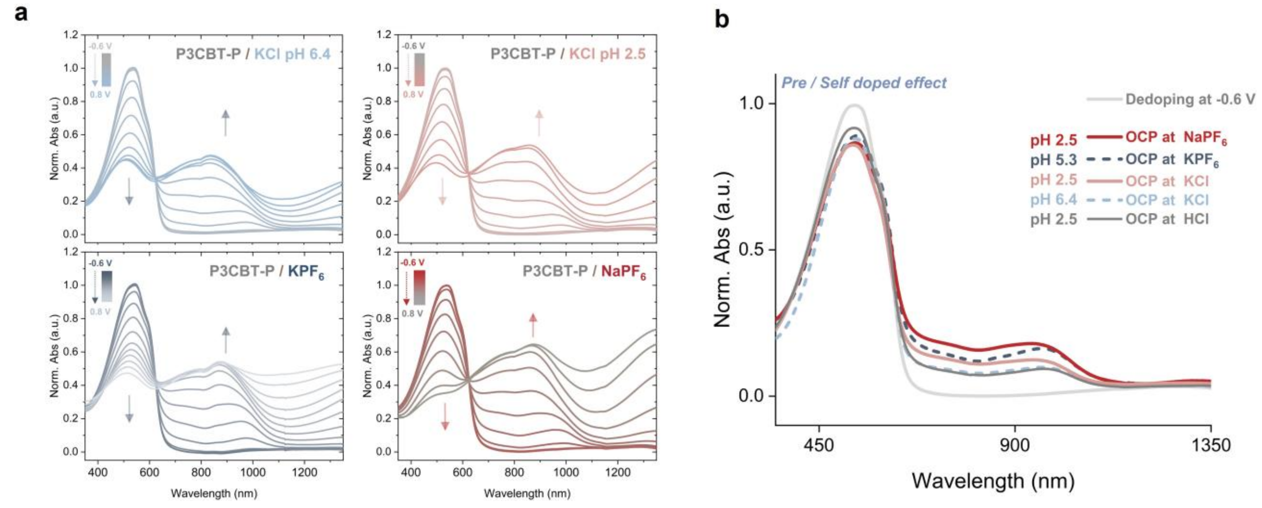

Figure S11: ***Operando*** **spectroelectrochemistry and pre- / self-doped evidence.** (a) Spectroelectrochemical response of P3CBT-P in pH-neutral electrolytes (0.1 M KCl and 0.1 M KPF_6, left) and pH-acidic electrolytes (KCl and $NaPF_6$, right). (b) UV–Vis spectra at open-circuit potential show polaronic absorption bands (800–1000 nm), indicating self-doping behavior. The intensity and position of these bands are both ion- and pH-dependent, with acidic conditions exhibiting a higher pre-doped state likely due to reduced cation–polymer interactions.

Transient absorption spectroscopy (TAS).

For samples of P3CBT-P under either neutral or acidic pH conditions, the results of the chirp correction to TAS data (2-panel figures) are shown below. A comparison of traces at 1000 nm are also presented to demonstrate the rate of the decay at both OCP and 0.8 V samples under neutral and acidic conditions. The TAS signal intensity is typically presented as a change in absorption (ΔA), which can broadly be understood as the difference between the excited-state absorption and the ground-state absorption. In cases when ΔA is relatively small, the change in absorption is denoted with a milli suffix (mΔA, in which mΔA $=10^{-3}$ ΔA units).

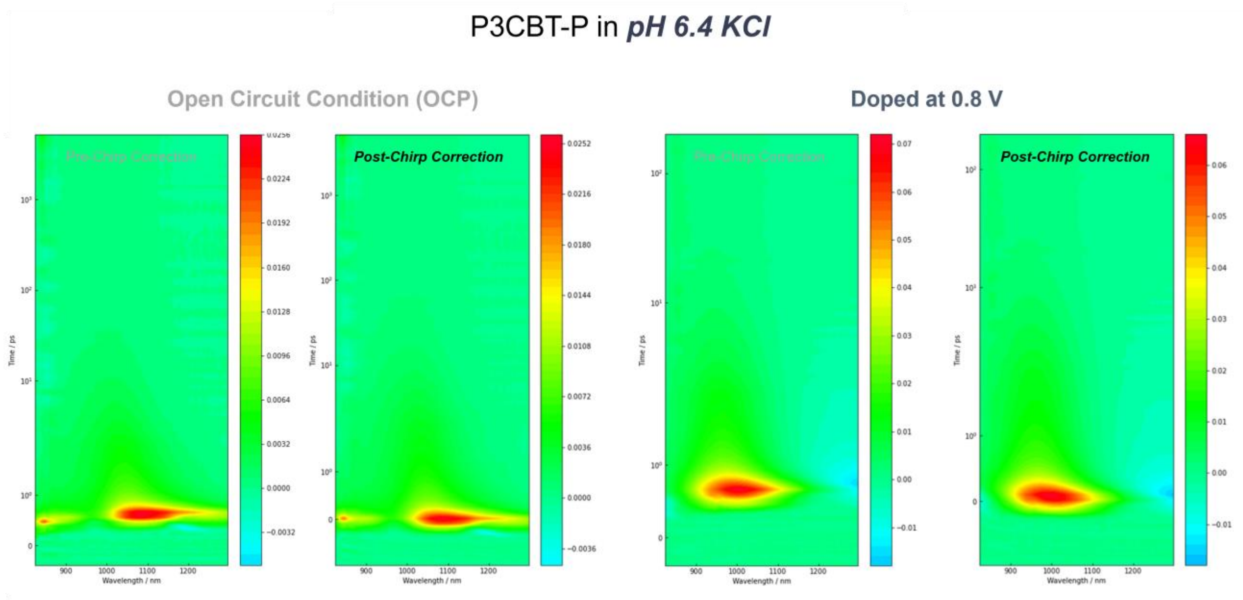

Figure S12: TAS raw data for carboxylated polythiophene under pH neutral condition. Raw data surface of the P3CBT-P sample in neutral pH 0.1 M KCl, shown at open-circuit potential (OCP, left) and under doping conditions (right), before and after chirp correction.

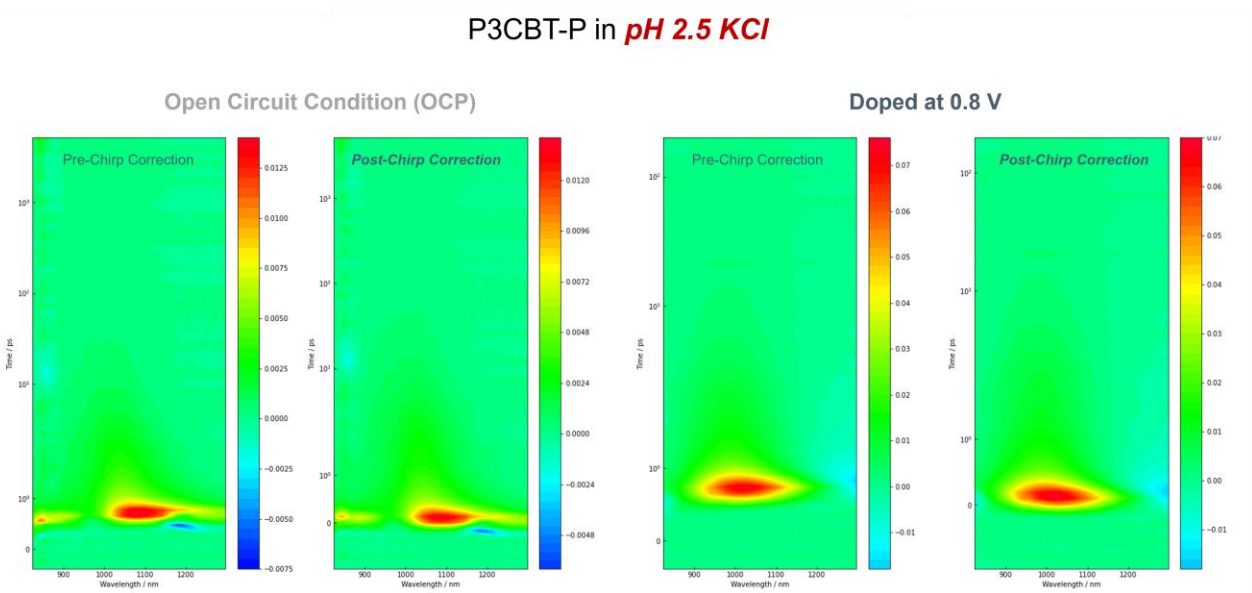

Figure S13: TAS raw data for carboxylated polythiophene under pH acidic condition. Raw data surface of the P3CBT-P sample in acidic pH 0.1 M KCl, shown at open-circuit potential (OCP, left) and under doping conditions (right), before and after chirp correction.

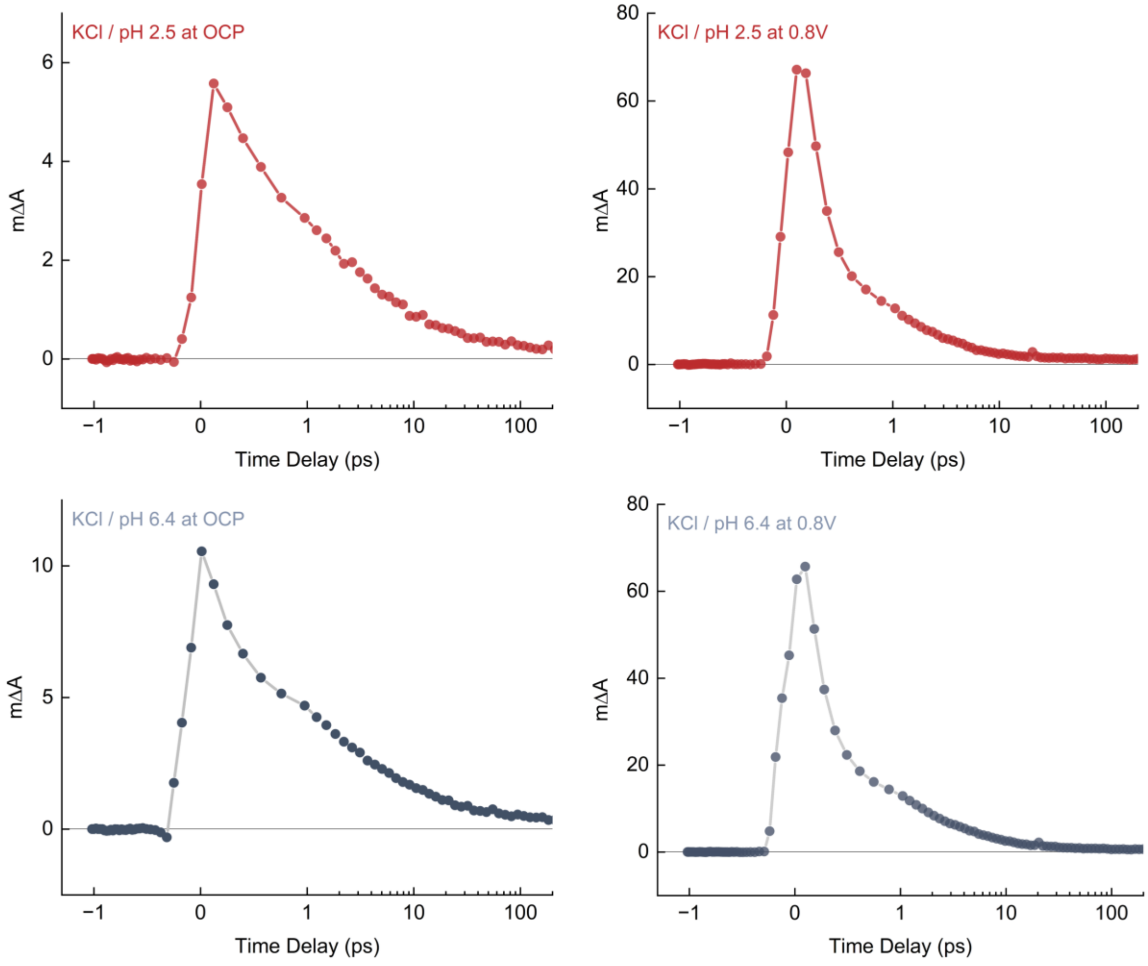

Figure S14: Kinetic trace at λ_{obs} = 1000 nm for TAS measurements on P3CBT-P under different conditions: OCP (top left), 0.8 V (top right) under pH acidic KCl conditions; and OCP (bottom left), and 0.8 V under pH neutral conditions (bottom right). Traces show the rate of decay for the TAS signal associated with the (polaron population) with much of the signal lost by ~10 ps after excitation at 850 nm.

Discussion 2: quantify fixed-charge density vs pH and identify dominating ionic species.

Open circuit potential (OCP) reflects the combined influence of ionic diffusion and interfacial potential, and it related to the diffusion potential by the equation: $E_{diffusion}$ = OCP – $E_{Redox.}$ E_{redox} term accounts for the unequal potential drop at the electrode-electrolyte interface.[16] To estimate the $E_{diffusion}$ term, we first measured the E_{redox} term, which captures the unequal potential drop at the electrode-electrolyte interface, using bare ITO electrolyte across various pH conditions in 0.1 M KCl. Subsequently, the OCP values were recorded using polymer coated ITO at the start of each CV cycle for 1 minute until stabilization. The calculated values are compiled in Table S1. The polarity of $E_{diffusion}$ indicates a dominant ionic species: negative values (< 0) suggest preferential anion diffusion, while the positive values (> 0) reflect cation dominance.

Unlike previously reported systems based on charge-neutral OMIECs, carboxylic acid functionalized polymers are conjugated polyelectrolytes, exhibiting pH-dependent fixed charge due to the ionizable nature of the side chains. And our main results have shown evidence of dual ion interaction during (de)doping cycles. At low pH (2.5), the polymer is mostly in its protonated (-COOH) from, resulting in minimal fixed charge and weak Donnan exclusion of anions. As pH increases, deprotonation leads to the accumulation of fixed negative charges (-COO-), progressively favoring cation uptake, and excluding anions. This transition is directly reflected in the measured diffusion potential as shown in Table, which shifts from negative values at low and neutral pH – indicative of anion-dominated ion gradient – to near-zero or even positive values at high pH, indicating a switch to cation-dominated diffusion.

*The anion contents within the film arises from both deprotonated carboxylates (COO-) and chloride ions in the electrolyte. At low pH, despite fewer COO- group due to higher protonation, stronger anion dominance is observed – likely due to increased Cl^- uptake resulting from weaker Donnan exclusion and reduced cation demand.

**Notably, the different behavior between P3CBT-P and P3CBT likely originate from processing factor, since P3CBT-P is derived from salt-form (COOK) polymer followed by acid treatment, while P3CBT is directly dissolved from its protonated form in DMSO. Previous work shows that P3CBT-P retains residual potassium, which can shift the Donnan potential and modify ion compensation. An additional contributing factor may be an intrinsic difference in acid content: P3CBT-P likely with a higher acid content (lower pKa), shows a full sign reversal in diffusion term by pH 9.7, while P3CBT only trend upward (higher pKa) – this behavior aligns with the expected pKa effect, as a more acidic polymer would indeed deprotonate more at a given pH, leading to a stronger Donnan potential and earlier crossover to the cation-dominance behavior. However, this hypothesis requires further experimental validation

Table S4: Qualitative interpretation of Open-Circuit Potentials in determining preference in OCP values for P3CBT-P and P3CBT in pH varied KCl electrolyte.

**P3CBT-P**	***pH 2.5***	***pH 6.4***	***pH 9.7***
OCP (V)	320 mV	200 mV	205 mV
E_{Redox} (V)	467 mV	333 mV	128 mV
$E_{Diffusion}$ (V)	**-147 mV**	**-133 mV**	**77 mV**

**P3CBT**	***pH 2.5***	***pH 6.4***	***pH 9.7***
OCP (V)	272 mV	188 mV	67 mV
E_{Redox} (V)	467 mV	333 mV	128 mV
$E_{Diffusion}$ (V)	**-195 mV**	**-145 mV**	**-61 mV**

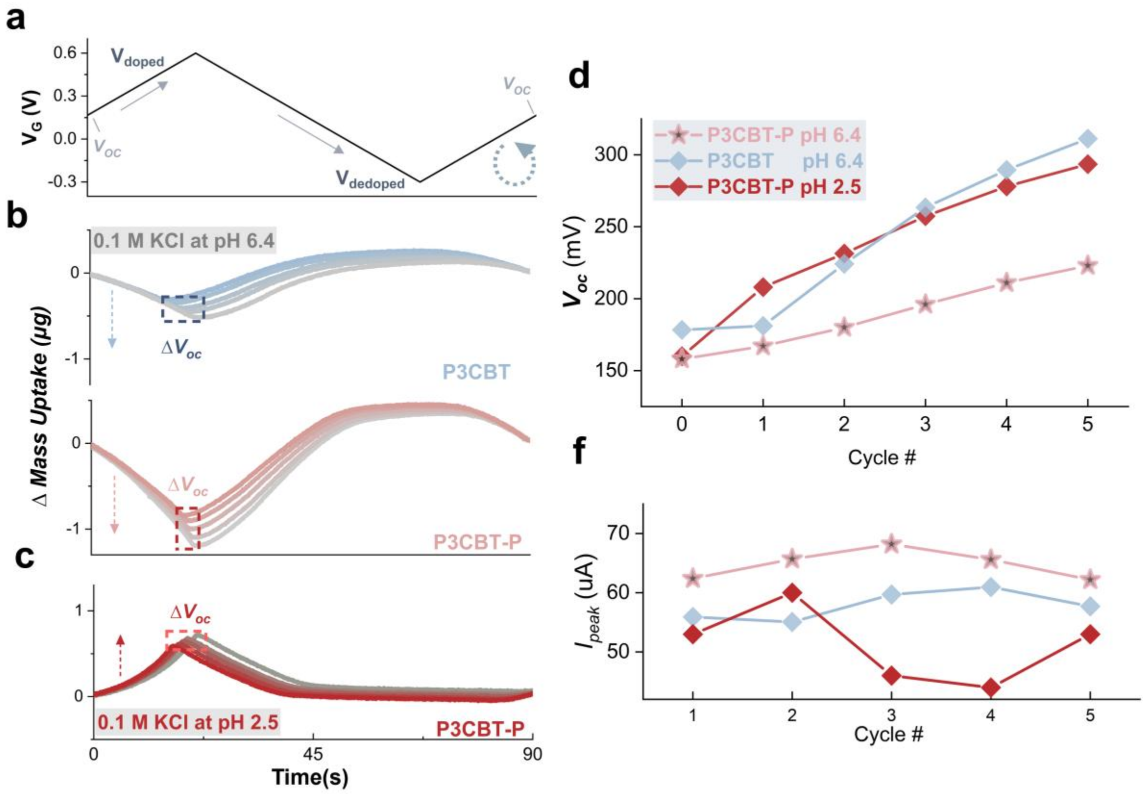

Figure S15: Open-circuit potential (OCP or V_{oc}) regulates ion uptakes in carboxylated mixed conductors. a) Experimental protocol: starting from OCP, the potential was cycled to 0.6 V, then to -0.3 V, and returned to OCP, repeated for 5 cycles with 20 second break periods between each cycle. b) Measurement for both P3CBT and P3CBT-P were conducted in 0.1 M KCl at pH neutral conditions. c) Measurement for P3CBT-P in 0.1 M KCl under acidic conditions. Since the OCP in acidic environments is typically higher than under neutral conditions, an initial preconditioning step was applied to lower the OCP to ~150 mV to enable a meaningful comparison. d) evolution of OCP, and e) peak current across cycles for all samples. The results show that both P3CBT in pH-neutral and P3CBT-P in pH-acidic conditions exhibit larger OCP drift across cycles, in contrast to the more stable behaviors of P3CBT-P at pH neutral. These findings suggest that strong cation-polymer interaction plays a functional role in stabilizing the OCP by buffering potential fluctuation during electrochemical cycling. The OCP may serve as an indicator of cation uptake and the extent of hydrophilic polymer-electrolyte interactions in carboxylated mixed conductors.

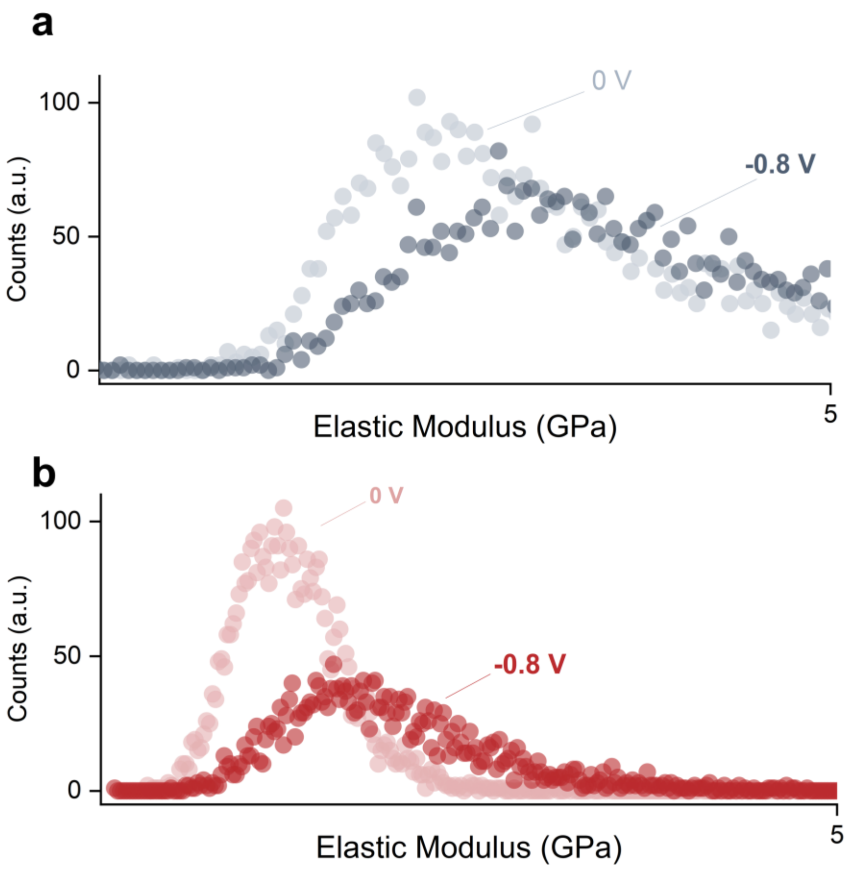

Figure S16: *Operando* force mapping revealing elastic modulus increase in a) P3CBT, and b) P3CBT-P during electrochemical doping at pH 3.5.

References:

1. Khot, A. & Savoie, B. M. How <scp>side-chain</scp> hydrophilicity modulates morphology and charge transport in mixed conducting polymers. *Journal of Polymer Science* 60, 610–620 (2022).

2. Marrink, S. J., Risselada, H. J., Yefimov, S., Tieleman, D. P. & de Vries, A. H. The MARTINI Force Field: Coarse Grained Model for Biomolecular Simulations. *J Phys Chem B* 111, 7812–7824 (2007).

3. Khot, A. & Savoie, B. M. Top–Down Coarse-Grained Framework for Characterizing Mixed Conducting Polymers. *Macromolecules* 54, 4889–4901 (2021).

4. Berardi, R., Fava, C. & Zannoni, C. A Gay–Berne potential for dissimilar biaxial particles. *Chem Phys Lett* 297, 8–14 (1998).

5. https://docs.lammps.org/pair_gromacs.html (accessed 2025-08-27). pair_style lj/gromacs command — LAMMPS documentation. .

6. Jing, L., Li, P., Li, Z., Ma, D. & Hu, J. Influence of π–π interactions on organic photocatalytic materials and their performance. *Chem Soc Rev* 54, 2054–2090 (2025).

7. Li, X. *et al.* Molecular Stacking Regulation of Organic Semiconductors Via Intermolecular Interaction Enables High-Performance Organic Solar Cells. *Adv Funct Mater* 35, (2025).

8. Modarresi, M., Franco-Gonzalez, J. F. & Zozoulenko, I. Morphology and ion diffusion in PEDOT:Tos. A coarse grained molecular dynamics simulation. *Physical Chemistry Chemical Physics* 20, 17188–17198 (2018).

9. Yesylevskyy, S. O., Schäfer, L. V., Sengupta, D. & Marrink, S. J. Polarizable Water Model for the Coarse-Grained MARTINI Force Field. *PLoS Comput Biol* 6, e1000810 (2010).

10. Thompson, A. P. *et al.* LAMMPS - a flexible simulation tool for particle-based materials modeling at the atomic, meso, and continuum scales. *Comput Phys Commun* 271, 108171 (2022).

11. Jewett, A. I. *et al.* Moltemplate: A Tool for Coarse-Grained Modeling of Complex Biological Matter and Soft Condensed Matter Physics. *J Mol Biol* 433, 166841 (2021).

12. Schmode, P. *et al.* Influence of ω-Bromo Substitution on Structure and Optoelectronic Properties of Homopolymers and Gradient Copolymers of 3-Hexylthiophene. *Macromolecules* 53, 2474–2484 (2020).

13. Rivnay, J., Mannsfeld, S. C. B., Miller, C. E., Salleo, A. & Toney, M. F. Quantitative Determination of Organic Semiconductor Microstructure from the Molecular to Device Scale. *Chem Rev* 112, 5488–5519 (2012).

14. Chae, S. *et al.* Impact of Molecular Weight on the Ionic and Electronic Transport of Self-Doped Conjugated Polyelectrolytes Relevant to Organic Electrochemical Transistors. *Adv Funct Mater* 34, (2024).

15. Sun, Z. *et al.* Controlling Ion Uptake in Carboxylated Mixed Conductors. *Advanced Materials* 37, (2025).

16. Ohayon, D. *et al.* Impact of Noncompensating Ions on the Electrochemical Performance of n-Type Polymeric Mixed Conductors. *J Am Chem Soc* 147, 12523–12533 (2025).
